# Four-dimensional nuclear speckle phase separation dynamics regulate proteostasis

**DOI:** 10.1126/sciadv.abl4150

**Published:** 2022-01-05

**Authors:** William Dion, Heather Ballance, Jane Lee, Yinghong Pan, Saad Irfan, Casey Edwards, Michelle Sun, Jing Zhang, Xin Zhang, Silvia Liu, Bokai Zhu

**Affiliations:** 1 Aging Institute of UPMC, University of Pittsburgh School of Medicine, Pittsburgh, PA, USA.; 2 UPMC Genome Center, Pittsburgh, PA, USA.; 3 Department of Chemistry, The Pennsylvania State University, University Park, PA, USA.; 4 Pittsburgh Liver Research Center, University of Pittsburgh, Pittsburgh, PA, USA.; 5 Department of Pathology, University of Pittsburgh School of Medicine, Pittsburgh, PA, USA.; 6 Division of Endocrinology and Metabolism, Department of Medicine, University of Pittsburgh; School of Medicine, Pittsburgh, PA, USA.

## Abstract

Phase separation and biorhythms control biological processes in the spatial and temporal dimensions, respectively, but mechanisms of four-dimensional integration remain elusive. Here, we identified an evolutionarily conserved XBP1s-SON axis that establishes a cell-autonomous mammalian 12-hour ultradian rhythm of nuclear speckle liquid-liquid phase separation (LLPS) dynamics, separate from both the 24-hour circadian clock and the cell cycle. Higher expression of nuclear speckle scaffolding protein SON, observed at early morning/early afternoon, generates diffuse and fluid nuclear speckles, increases their interactions with chromatin proactively, transcriptionally amplifies the unfolded protein response, and protects against proteome stress, whereas the opposites are observed following reduced SON level at early evening/late morning. Correlative *Son* and proteostasis gene expression dynamics are further observed across the entire mouse life span. Our results suggest that by modulating the temporal dynamics of proteostasis, the nuclear speckle LLPS may represent a previously unidentified (chrono)-therapeutic target for pathologies associated with dysregulated proteostasis.

## Teaser

A cell-autonomous 12-hour clock coordinates nuclear speckle LLPS with proteostasis control.

## INTRODUCTION

Most life on earth is governed by biological rhythms that are defined as self-sustained oscillations cycling with a fixed period. Biological clocks enable organisms to keep track of the time of day and to adjust their physiology to recurring daily changes in the external environment, including nutrient and microenvironment status. Our understandings of biological rhythms in mammals have expanded beyond the well-characterized circadian rhythms (~24-hour oscillation) in recent years through the discovery of 12-hour ultradian rhythms in mammals (*1*, *2*). In contrast to earlier hypothesis that these 12-hour rhythms are under the combined regulation of the 24-hour circadian clock and feeding/fasting cues (*2*, *3*), our group identified a cell-autonomous mammalian 12-hour ultradian oscillator that regulates 12-hour rhythms of systemic gene expression and metabolism (*4*). The 12-hour oscillator is independent from the 24-hour circadian clock but instead is regulated by the unfolded protein response (UPR) transcription factor spliced form of X-Box Binding Protein 1 (XBP1s) (*4*–*7*). In mouse, the liver-specific deletion of XBP1s impaired more than 80% of 12-hour transcriptome, while leaving the majority of circadian transcriptome intact (including all known core circadian clock genes) (*5*, *6*). As a result of the 12-hour clock ablation, XBP1s liver-specific knockout (XBP1*^LKO^*) mice exhibited markedly accelerated liver aging and fatty liver diseases (*5*).

Subsequent Gene Ontology (GO) analysis of XBP1s-dependent mouse hepatic 12-hour transcriptome revealed top-enriched genes involved in the entire central dogma information flow (CEDIF) process, ranging from transcription initiation, mRNA processing and export, ribosome biogenesis, translation initiation to protein folding, processing and sorting in the endoplasmic reticulum (ER) and Golgi, as well as various metabolic pathways including lipid and nucleotide metabolism (*6*). While the regulation of protein and lipid homeostasis by XBP1s is well established (*8*), the control of mRNA metabolism by XBP1s and the mechanistic link between mRNA and protein homeostasis remain poorly characterized. Therefore, in this study, we aim to uncover the underlying mechanisms of coordinated mRNA and protein metabolism by investigating the 12-hour oscillator. By combining single-cell time-lapse microscopy, cistrome profiling, and mathematical modeling, we unexpectedly identified an XBP1s-SON axis that dictates a cell-autonomous mammalian 12-hour ultradian rhythm of nuclear speckle liquid-liquid phase separation (LLPS) dynamics, which drives rhythmic global 12-hour nuclear speckle–chromatin interactions, uncoupled from the transcriptional state of individual genes. We found that the expression of genes involved in proteostasis, including *Xbp1* itself, is hypersensitive to nuclear speckle LLPS dynamics change. We further observed correlative *Son* and proteostasis gene expression dynamics during the transient response to ER stress and across the entire mouse life span. Functionally, the XBP1s-SON axis can protect cells from proteome stress via transcriptionally amplifying the UPR. Our results thereby uncovered an intrinsic feedforward loop connecting nuclear speckle LLPS and proteostasis control that likely ensures a highly efficient genetic information transfer functioning at multiple temporal scales.

## RESULTS

### XBP1s regulates a cell-autonomous 12-hour rhythm of nuclear speckle morphology change

We first confirmed that the 12-hour rhythms of mRNA metabolic genes are also observed at the protein level by performing a post hoc analysis of two published hepatic nuclear protein mass spectrometry datasets (fig. S1, A and B, and table S1) (*9*, *10*). GO analysis of 12-hour nuclear proteins in both datasets revealed top enriched GO term “spliceosome” (fig. S1C). Spliceosome is predominantly found in nuclear speckles, which are membraneless organelles enriched in pre-mRNA processing factors, as well as various other proteins and noncoding RNAs (ncRNAs) involved in RNA export, transcription regulation, pre-mRNA cleavage and polyadenylation, and RNA degradation (*11*, *12*). We further observed a robust 12-hour expression of nuclear speckle ncRNA *Malat1*, which is also a direct transcriptional target gene of XBP1s and exhibits slightly dampened rhythm in XBP1 liver-specific knockout (XBP1*^LKO^*) mice from our previously published RNA sequencing (RNA-seq) dataset (fig. S1, D and E) (*6*).

**Fig. 1.**
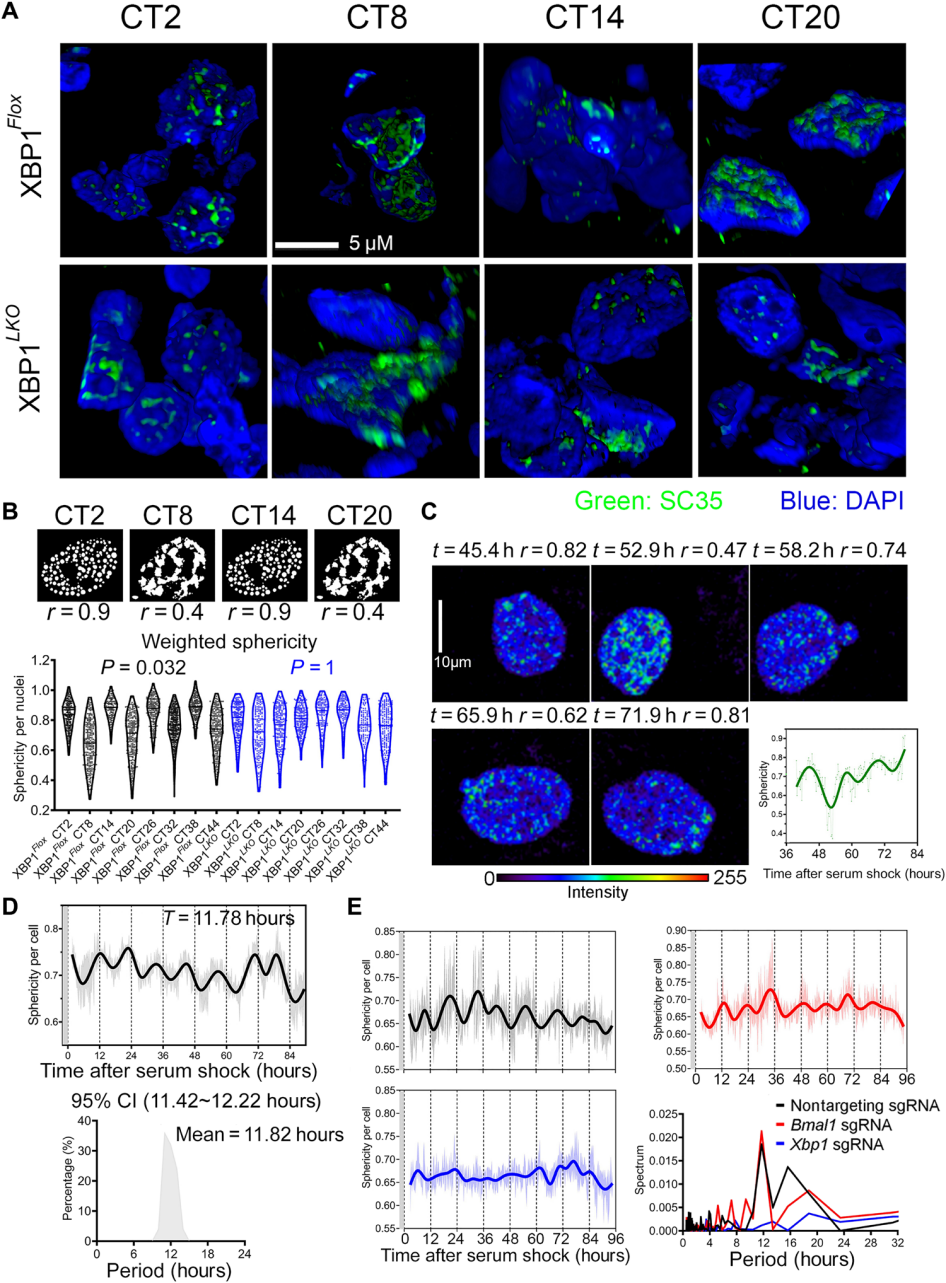
XBP1s regulates a cell-autonomous 12-hour rhythm of nuclear speckle morphology change. (**A**) Three-dimensional reconstruction of immunofluorescence of SC35 costained with 4′,6-diamidino-2-phenylindole (DAPI) in the liver of XBP1*^Flox^* and XBP1*^LKO^* mice at different CTs. (**B**) Cartoon showing the different nuclear speckle LLPS patterns at different CTs in XBP1*^Flox^* mice (top) and violin plot quantification of weighted sphericity of nuclear speckles in XBP1*^Flox^* and XBP1*^LKO^* mice liver at different CTs. *n* = 150 to 400 nuclei from three mice per CT. *P* values exhibiting statistically significant 12-hour rhythms by RAIN analysis in XBP1*^Flox^* and XBP1*^LKO^* mice are also shown. (**C** to **E**) GFP::SC35 mouse embryonic fibroblasts (MEFs) were serum synchronized and subject to time-lapse imaging. Selective images and quantification of temporal sphericity from one single GFP::SC35 MEF. Narrow line, raw data; thick line, spline fit (C). Temporal sphericity (6 to 10 cells quantified at any given time; gray area, means ± SEM; solid line, spline fit) and period distribution of dominant sphericity rhythms in single cells quantified by the eigenvalue/pencil method (*n* = 25) (D). Temporal sphericity of GFP::SC35 MEFs expressing control, *Bmal1*, or *Xbp1* single-guide RNA (sgRNA) (6 to 10 cells quantified at any given time; light area, means ± SEM; solid line, spline fit), and periodogram analysis of average sphericity rhythms in the three groups (E). Gray areas in (D) and (E) indicate 2 hours of serum shock.

To look for additional evidence of 12-hour mRNA metabolism besides gene expression, we initially asked whether the morphologies of nuclear speckle may exhibit time-of-the-day variation. Nuclear speckles normally are microscopically presented as “punctate” nuclear localization pattern, formed through LLPS because of the prevalence of low complexity domains found in splicing factors (*11*–*13*). We performed immunofluorescence against SC35 (SRSF2), one well-established marker of nuclear speckles (*12*, *13*), in the liver section of XBP1*^Flox^* and XBP1*^LKO^* mice at different circadian time (CTs). Consistent with previous observations (*12*, *14*), SC35-positive loci are associated with particularly low chromatin density, but in close proximity to chromatin, at all times in both XBP1*^Flox^* and XBP1*^LKO^* mice (fig. S2A). Nonetheless, we observed markedly distinct staining patterns of SC35 in XBP1*^Flox^* mouse liver at different times of day. At CT2, CT14, CT26, and CT38, spherical punctate patterns of SC35 found in the majority of nuclei are suggestive of LLPS formed via a process termed “nucleation” (*15*). By contrast, at CT8, CT20, CT32, and CT44, we observed a much more diffuse and network-like spatial distribution of SC35 staining, reminiscent of LLPS formed via spinodal decomposition (Fig. 1A and fig. S2A) (*15*). Quantifying these two distinct forms of LLPS by calculating the roundness of nuclear speckle staining as previously described (*16*) evinced an apparent 12-hour rhythm of nuclear speckle LLPS dynamics in XBP1*^Flox^* [*P* value of 0.032 by Rhythmicity Analysis Incorporating Nonparametric (RAIN) analysis (*17*)], but not in XBP1*^LKO^* mice (*P* value of 1 by RAIN analysis) (Fig. 1B), which is further confirmed by staining against a different nuclear speckle marker SON (*P* value of 0.031 and 0.54 in XBP1*^Flox^* and XBP1*^LKO^* mice by RAIN analysis, respectively) (fig. S2B).

To determine whether the 12-hour rhythm of nuclear speckle morphology change is cell autonomous, we used CRISPR-CAS9 system to knock in green fluorescent protein (GFP) to the N-terminal region of endogenous SC35 locus in immortalized mouse embryonic fibroblasts (MEFs) and performed time-lapse imaging to track nuclear speckle morphology changes in single cells over time. This system allows us to probe nuclear speckle phase separation dynamics under physiological condition without the overexpression of speckle proteins. Western blot and immunofluorescence confirmed the successful generation of MEFs expressing GFP::SC35 fusion protein (fig. S2, C and D). In MEF synchronized by serum shock, we observed robust in-phase ~12-hour rhythms of nuclear speckle morphology changes alternating between a high roundness punctate state and a low roundness diffuse state in single cells (Fig. 1, C and D, fig. S2E, and movie S1). However, consistent with our previous study (*4*), this 12-hour rhythm of nuclear speckle morphology changes is independent from the cell cycle (fig. S2, F and G). In unsynchronized MEFs (no prior serum shock), we observed a weak local synchronization for cells within 140 μm of each other, in terms of their nuclear speckle morphology oscillation (fig. S3, A to H), implying the very likely existence of paracrine factors for the local synchronization of 12-hour oscillator in adjacent cells in vitro.

Next, we used CRISPR-CAS9 to knock out the circadian clock master regulator BMAL1, or XBP1 in GFP::SC35 MEFs (fig. S4, A to C), and found that XBP1, but not BMAL1, is required for the establishment of the 12-hour rhythm of nuclear speckle morphology change (Fig. 1E). Because the ER-localized endoribonuclease IRE1α can act both upstream (via alternatively splicing *Xbp1* mRNA to generate *Xbp1s*) and downstream (*Ire1*α mRNA exhibits XBP1s-dependent 12-hour rhythm) of XBP1s and was previously proposed to be an integral component of 12-hour oscillator regulatory network (fig. S5, A to C) (*7*), we further investigated whether IRE1α inhibition can also impair the 12-hour rhythm of nuclear speckle morphology change. Treating GFP::SC35 MEFs with the selective IRE1α inhibitor 4μ8c resulted in a low-amplitude ~8-hour oscillation instead (fig. S5D). In summary, we therein demonstrated an IRE1α/XBP1s-dependent cell-autonomous 12-hour ultradian rhythm of nuclear speckle morphology that alternates between a more punctate and a more diffuse state. This 12-hour ultradian rhythm is further uncoupled from both the cell cycle and the 24-hour circadian clock and exhibits local coupling in otherwise globally unsynchronized cells.

### An evolutionarily conserved XBP1s-SON axis controls 12-hour rhythm of cell-autonomous nuclear speckle LLPS dynamics

**Fig. 2.**
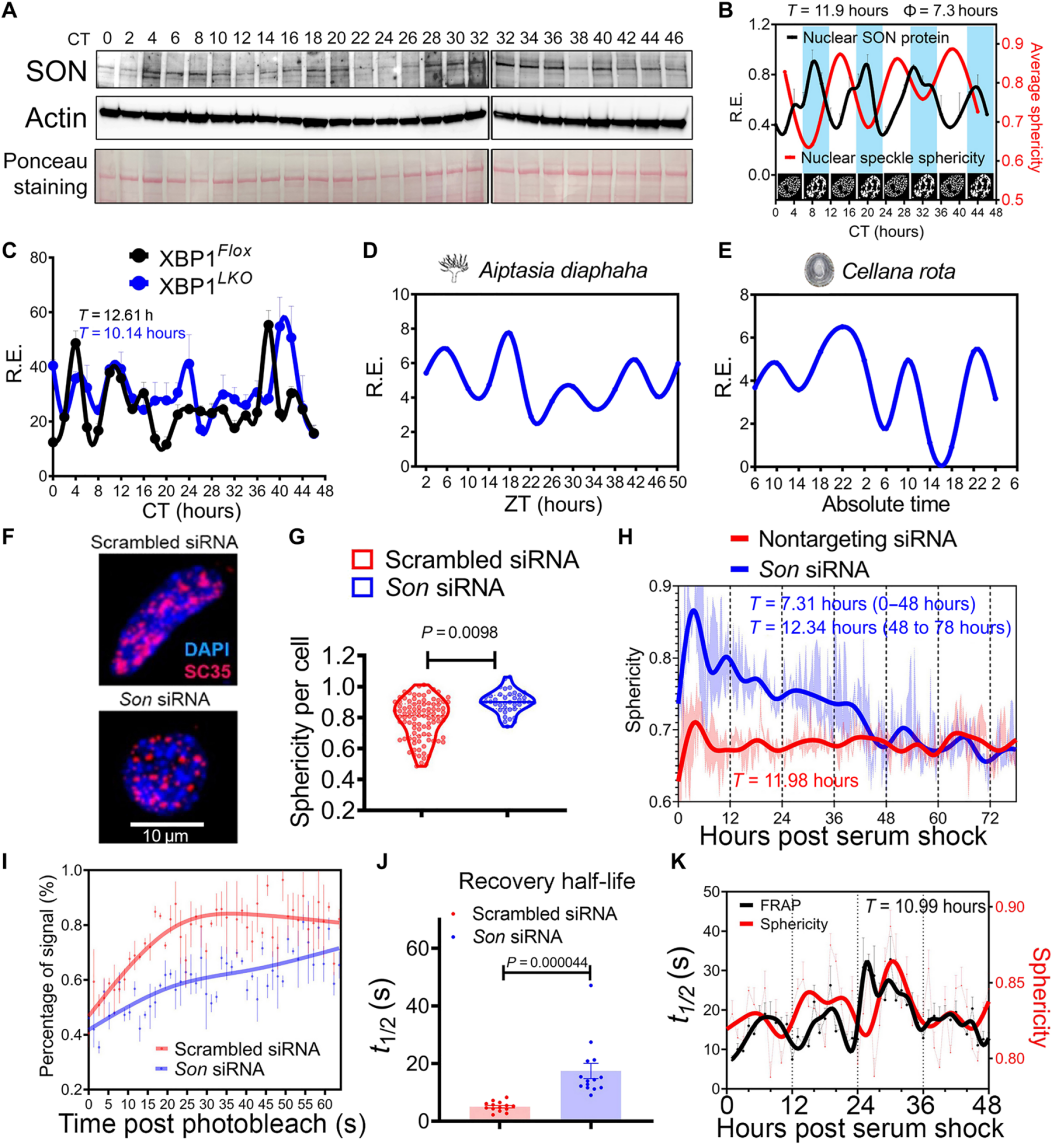
An evolutionarily conserved XBP1s-SON axis controls 12-hour rhythm of cell-autonomous nuclear speckle LLPS dynamics. (**A** and **B**) Representative Western blot (A) and quantification (B) (*n* = 2 to 4) of hepatic nuclear SON protein (normalized to the Ponceau S staining intensity), superimposed with the average nuclear speckle sphericity and morphology cartoons at different CTs. The same CT32 sample was run twice to enable comparison across different gels. (**C**) qPCR analysis of hepatic *Son* in in the liver of XBP1*^Flox^* and XBP1*^LKO^* mice at different CTs. Periods calculated by the eigenvalue/pencil method are also shown. (**D** and **E**) RNA-seq data of *Son* ortholog expression in *A. diaphaha* (D) and *C. rota* (E). (**F** and **G**) Immunofluorescence of SC35 costained with DAPI in scrambled or *Son* siRNA MEFs (F) and quantification of sphericity (*n* = 40 to 100) (G). (**H** to **J**) GFP::SC35 MEFs were transfected with nontargeting scrambled or *Son* siRNA. Temporal sphericity (5 to 10 cells quantified at any given time; light area, means ± SEM; solid line, spline fit) and calculated periods by the eigenvalue/pencil method after serum synchronization (H). FRAP analysis with representative recovery curve (I) (data showing quantification from three speckles per cell; means ± SEM for each point; solid line, spline fit) and quantified recovery half-life (J). (**K**) Calculated temporal sphericity and recovery half-life of nuclear speckles from serum synchronized GFP::SC35 MEFs. *n* = 7 to 10 cells for FRAP analysis; sphericity was calculated from the average sphericity of each image (*n* = 6) with ~20 nuclear speckles on each image that are captured before photobleaching. Dash line, raw data; solid line, spline fit.

Thus, what may be the mechanism(s) underlying the observed 12-hour rhythm of nuclear speckle morphology change? A recent study on LLPS demonstrated that the concentration of scaffolding protein in the condensates can dictate the way by which LLPS occurs (*15*, *18*). Simply put, under constant valency condition, a higher concentration of scaffolding protein (within the blue region of the phase diagram) will induce LLPS via spinodal decomposition (thus more diffuse), while a lower concentration (within the red region of the phase diagram) will lead to nucleation (thus rounder) (fig. S6, A and B). In nuclear speckles, the protein SON was previously hypothesized to act as a scaffolding protein upon which other RNA processing factors and ncRNA assemble (*19*). To test the idea that SON level may dictate nuclear speckle morphology, we first performed Western blot analysis to examine the nuclear level of SON in the liver of wild-type mice at different times of day and observed a robust 12-hour oscillation of SON nuclear expression peaking at ~CT8, CT20, CT32, and CT44 (Fig. 2, A and B, and fig. S6C). The oscillation of SON is antiphase with that of nuclear speckle sphericity, and the amplitude of the physiological level of SON oscillation is two- to threefold change (Fig. 2, A and B). Both the phase and amplitude of 12-hour SON oscillation are consistent with the theoretical prediction by the LLPS phase diagram (Fig. 2, A and B, and fig. S6, A to C). The 12-hour rhythm of hepatic SON expression is further validated by the hepatic mass spectrometry dataset (fig. S6, D and E) (*9*). We next performed quantitative polymerase chain reaction (qPCR) and identified a 12-hour rhythm of *Son* expression at the mRNA level in XBP1*^Flox^* mice (Fig. 2C and fig. S6F). By contrast, the period of *Son* mRNA oscillation was shortened to ~10-hour in XBP1*^LKO^* mice (Fig. 2C and fig. S6F). Cell-autonomous 12-hour *Son* mRNA expression is further identified in serum synchronized MMH-D3 hepatocytes in vitro (*20*), maintaining a similar relative phase to that of *Bmal1* as in vivo (fig. S6, G and H).

To determine whether *Son* is a direct transcriptional target gene of XBP1s, we first examined our previously published hepatic XBP1s chromatin Immunoprecipitation sequencing (ChIP-seq) dataset (*6*) and found a 12-hour XBP1s chromatin recruitment to the *Son* gene promoter region (fig. S6I). Consistent with the ChIP-seq result, motif analysis scanning a 1-kb region of the *Son* gene promoter further identified the XBP1s consensus binding sequence ACGTCA (fig. S6J). In addition, transient overexpression of XBP1s increased the expression of *Son* mRNA, together with canonical UPR genes *Manf* and *Hyou1* (fig. S6K). While these results strongly indicate that *Son* is under the direct transcriptional control of XBP1s, it is highly likely that additional transcriptional factors also regulate *Son* gene expression. Our motif analysis also uncovered putative binding sites for GABPA and NFYA, two additional transcriptional factors involved in the 12-hour oscillator control (fig. S6J) (*Gabpa* and *Nyfa* themselves are also direct XBP1s transcriptional target genes) (*7*). The potential involvement of multiple interlocked transcriptional loops in the regulation of *Son* gene expression is also likely responsible for its shortened ~10-hour period observed in XBP1*^LKO^* mice, as previously seen in circadian period control (*21*).

We previously proposed that the mammalian 12-hour clock likely evolved from the ancient circatidal clock of marine animals, who adapt their behaviors to the ~12-hour ebb and flow of the tides resulting from the gravitational pull of the moon (*1*, *22*). Evidence supporting this evolutionary origin includes conserved 12-hour rhythms of gene expression between mice and two marine species: the sea anemone *Aiptasia diaphana* and the limpet *Cellana rota* (*6*). In line with this evolutionary conservation, we observed robust 12-hour rhythms of mRNA expression of *Son* orthologs in *A. diaphana* (*23*) and *C. rota* (Fig. 2, D and E) (*24*). mRNA processing is the most enriched biological pathway associated with circatidal genes in both marine animals (*6*). Because the 12-hour rhythm of *Xbp1* expression was previously found to be also conserved in marine species (*1*), these results indicate that the 12-hour rhythm of the XBP1s-SON axis is evolutionarily conserved.

To establish the causality between SON expression and nuclear speckle morphology, we knocked down or overexpressed SON by small interfering RNA (siRNA) or the CRISPR-dCAS9-VPR (VP64-p65-Rta) (*25*) system in GFP::SC35 MEFs, respectively (fig. S7, A to F). As expected, both manipulations impaired the 12-hour rhythm of cell-autonomous nuclear speckle morphology change, with the former increasing (Fig. 2, F to H, and fig. S7C) and the latter decreasing (fig. S7G) the average roundness of nuclear speckles, again in line with the theoretical prediction by the LLPS phase diagram. This morphology change is ostensibly reversible, as in the later days of imaging, the 12-hour rhythm of nuclear speckle morphology change is restored, likely due to the gradual loss/dilution of *Son* siRNA (Fig. 2H). To determine whether the morphology changes of nuclear speckle are associated with fluctuations in its dynamics, we performed fluorescence recovery after photobleaching (FRAP) in GFP::SC35 MEFs and found that SON positively regulates the fluidity of nuclear speckles (Fig. 2, I and J; fig. S7, H and I; and movies S2 and S3). We further observed a very robust 12-hour rhythm (with a range of ~8 to ~32 s of recovery half-life) of nuclear speckle fluidity in serum shock–synchronized GFP::SC35 MEFs, well in line with the 12-hour rhythm of nuclear speckle sphericity, with decreased fluidity (longer recovery half-life) coinciding with rounder nuclear speckles (sphericity approaching 1) (Fig. 2K). Thus far, we have demonstrated an evolutionarily conserved XBP1s-SON axis that controls a 12-hour rhythm of cell-autonomous nuclear speckle LLPS dynamics: A higher level of SON expression, observed at early afternoon (CT8) and early morning (CT20), leads to a more diffuse and fluid nuclear speckle morphology, while a lower SON expression, seen at early evening (CT14) and late morning (CT2), renders nuclear speckles to a more spherical and more stagnant state.

### XBP1s regulates 12-hour rhythmic nuclear speckle–chromatin interactions

**Fig. 3.**
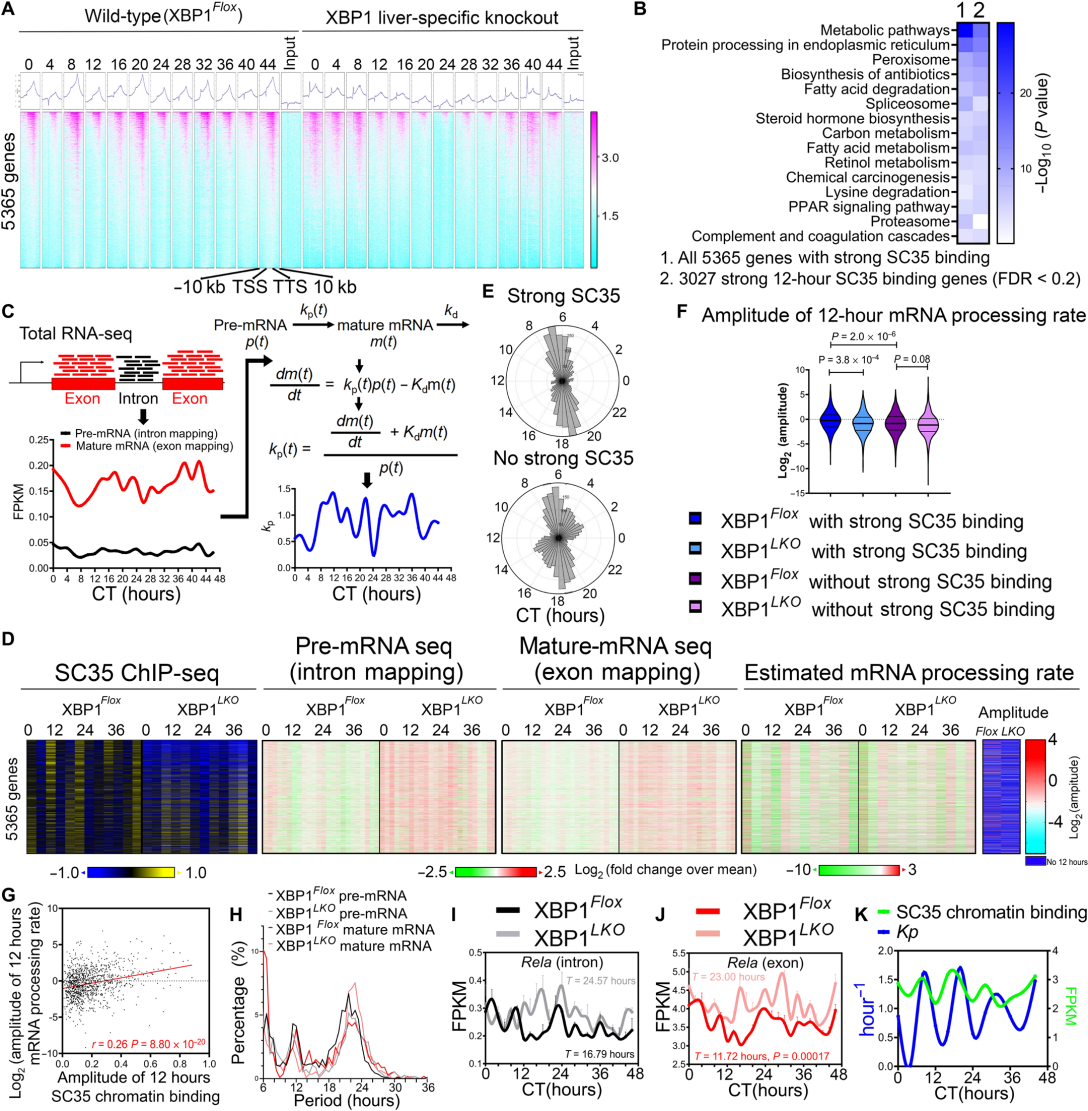
XBP1s regulates 12-hour rhythmic nuclear speckle–chromatin interactions. (**A**) Heatmap of temporal SC35 ChIP-seq signal as well as input signal for 5365 genes in XBP1*^Flox^* and XBP1*^LKO^* mice from 10 kb upstream of transcription start site (TSS) to 10 kb downstream of transcription termination site (TTS) for each gene. (**B**) GO analysis of all 5365 genes or 3207 genes with strong SC35 ChIP signal. PPAR, peroxisome proliferator–activated receptor. (**C**) Illustration on the workflow to estimate the pre-mRNA and mature-mRNA level, as well the as the mRNA processing rate from temporal RNA-Seq data. (**D**) Heatmaps of relative integrated SC35 binding signal over gene bodies, pre-mRNA and mature-mRNA expression, and estimated mRNA processing rate (with amplitude for 12-hour rhythm) at different CTs in XBP1*^Flox^* and XBP1*^LKO^* mice. (**E**) Polar histogram demonstrating the phase distributions of 12-hour rhythmic mRNA processing rates for genes with (top) or without (bottom) strong SC35 signal in XBP1*^Flox^* mice. (**F**) Log_2_-transformed amplitude of 12-hour mRNA processing rates for genes with or without strong SC35 binding in XBP1*^Flox^* and XBP1*^LKO^* mice. (**G**) Scatter plot of the amplitude of log_2_-transformed 12-hour mRNA processing rates versus integrated 12-hour SC35 binding signal over gene bodies for 1160 genes having both in XBP1*^Flox^* mice. (**H**) Period distribution of pre-mRNA and mature-mRNA oscillations for 1160 genes in (G). (**I** to **K**) Temporal expression of *Rela* at the pre-mRNA (I) and mature-mRNA (J) level in XBP1*^Flox^* and XBP1*^LKO^* mice, and integrated SC35 gene body signal and mRNA processing rate in XBP1*^Flox^* mice (K). Data are means ± SEM in (I) and (J).

Next, we wondered whether these observed nuclear speckle condensates dynamics lead to a change in their spatial distribution, more specifically, their propensity to associate with chromatin. To detect temporal nuclear speckle–chromatin interactions, we performed ChIP-seq in the liver of XBP1*^Flox^* and XBP1*^LKO^* mice at 4-hour interval for 2 days at constant darkness, using a monoclonal antibody against mouse SC35 (SRSF2), which has recently been successfully used to characterize splicing condensate–chromatin interactions (*26*). Consistent with previous SC35 ChIP-seq and *Malat1* Capture hybridization analysis of RNA targets (CHART)–seq results (*26*, *27*), nuclear speckle–chromatin interactions are enriched in gene bodies, with a gradual increase in binding intensity toward the 3′ transcription termination site (TTS), as exemplified by very strong binding observed at *Neat1* and *Malat1* themselves as expected (Fig. 3A and fig. S8A). We observed a dominant global 12-hour rhythm of nuclear speckle–chromatin interactions in XBP1*^Flox^* mice cresting at CT8, CT20, CT32, and CT44 (Fig. 3A and fig. S8, A to F), which corresponds to peaking SON expression and nuclear speckle fluidity and diffuseness at the same time (Figs. 1, A and B, and 2, A and B). We identified a total of 5365 genes in wild-type mice that have high-confidence nuclear speckle–chromatin interactions within gene bodies, and 3027 of them have robust ~12-hour rhythmic nuclear speckle–chromatin interaction [with a false discovery rate (FDR) cutoff of 0.2 by RAIN analysis] (Fig. 3A and tables S2 and S3). These genes are very strongly enriched in metabolic and protein homeostasis/ER stress pathways (Fig. 3B). Although below detection threshold for peak calling algorithms, weaker 12-hour rhythmic nuclear speckle–chromatin interactions were also observed in an additional 4130 genes by the eigenvalue/pencil method (table S4 and fig. S8, B, D, and F) (*4*, *28*), which together with the 3027 genes account for more than half of all expressed genes in the wild-type mouse liver. In contrast, ~12-hour rhythms of global nuclear speckle–chromatin interactions were substantially impaired in XBP1*^LKO^* mice. Instead, a low-amplitude ~10-hour global binding rhythm was observed after polynomial detrend (Fig. 3A; fig. S8, A to G; and table S4). Notably, this rhythmicity is in line with the ~10-hour *Son* mRNA oscillation observed in XBP1*^LKO^* mice liver (Fig. 2C and fig. S6F).

To determine whether the rhythmic nuclear speckle–chromatin interactions are correlated with transcriptional state fluctuations in mouse liver in vivo, we estimated the temporal pre-mRNA and mature-mRNA level of each hepatic gene by, respectively, quantifying the reads mapped to intron and exon regions, using our previously published RNA-seq dataset (*6*) (Fig. 3C and tables S5 and 6). Consistent with past studies (*12*, *13*, *29*), on a global scale, higher daily-average nuclear speckle–chromatin recruitment is strongly associated with higher daily-average gene expression in both XBP1*^Flox^* and XBP1*^LKO^* mice (fig. S9A). However, on an individual gene level, temporal rhythmic nuclear speckle–chromatin interaction is largely decoupled from temporal gene expression, as not all nuclear speckle–associated genes exhibit 12-hour rhythms of expression (Fig. 3D). We further estimated the mRNA processing rate of each gene via a simple first-order kinetic model of transcription regulation (*30*) (assuming the mRNA degradation rate remains constant during a day) and found a strong positive correlation between 12-hour rhythmic nuclear speckle–chromatin association and 12-hour rhythmic mRNA processing rate in XBP1*^Flox^* mice that both peak around CT8/CT20 (Fig. 3, C to G, and fig. S9B). Compared to XBP1*^Flox^* mice, the daily-average mRNA processing rate in XBP1*^LKO^* mice liver was slightly reduced (fig. S9C) and exhibited a dominant population of shortened ~10-hour oscillations (Fig. 3D and fig. S9D), again in line with observed ~10-hour rhythm of *Son* expression (Fig. 2C and fig. S6F) and ~10-hour oscillation of nuclear speckle–chromatin interactions (fig. S8, F and G). For those genes that do maintain ~12-hour mRNA processing rate in XBP1*^LKO^* mice, they have lower amplitude compared with their wild-type counterparts (Fig. 3F) and a more diffuse phase distribution (fig. S9E). Twelve-hour rhythmic nuclear speckle–chromatin interaction and subsequent 12-hour mRNA processing rates greatly contribute to the establishment of 12-hour rhythm, but not 24-hour circadian rhythm of gene expression posttranscriptionally in XBP1*^Flox^* mice (Fig. 3H), with examples of *Rela* and *Id1* genes only exhibiting 12-hour rhythms at the mature-mRNA level in XBP1*^Flox^* mice (Fig. 3, I to K, and fig. S9, F to H). Dominant 24-hour circadian rhythms of nuclear speckle–chromatin interactions and mRNA processing rates were observed on all core circadian clock genes with similar amplitudes in both XBP1*^Flox^* and XBP1*^LKO^* mice, in phase to their respective temporal gene expression profile (fig. S10, A to D), although some genes (such as *Per1* and *Nfil3*) have weaker superimposed 12-hour rhythmic nuclear speckle–chromatin interactions (also peaking at CT8/CT20), which are lost in XBP1*^LKO^* mice (fig. S10C). Because the core circadian gene expressions are not altered in XBP1*^LKO^* mice (*6*), these results suggest that the SON-mediated 12-hour rhythmic nuclear speckle–chromatin interactions are largely dispensable for the establishment of 24-hour core circadian clock gene expression in mice.

### Proteostasis gene expressions are hypersensitive to nuclear speckle LLPS dynamic change

**Fig. 4.**
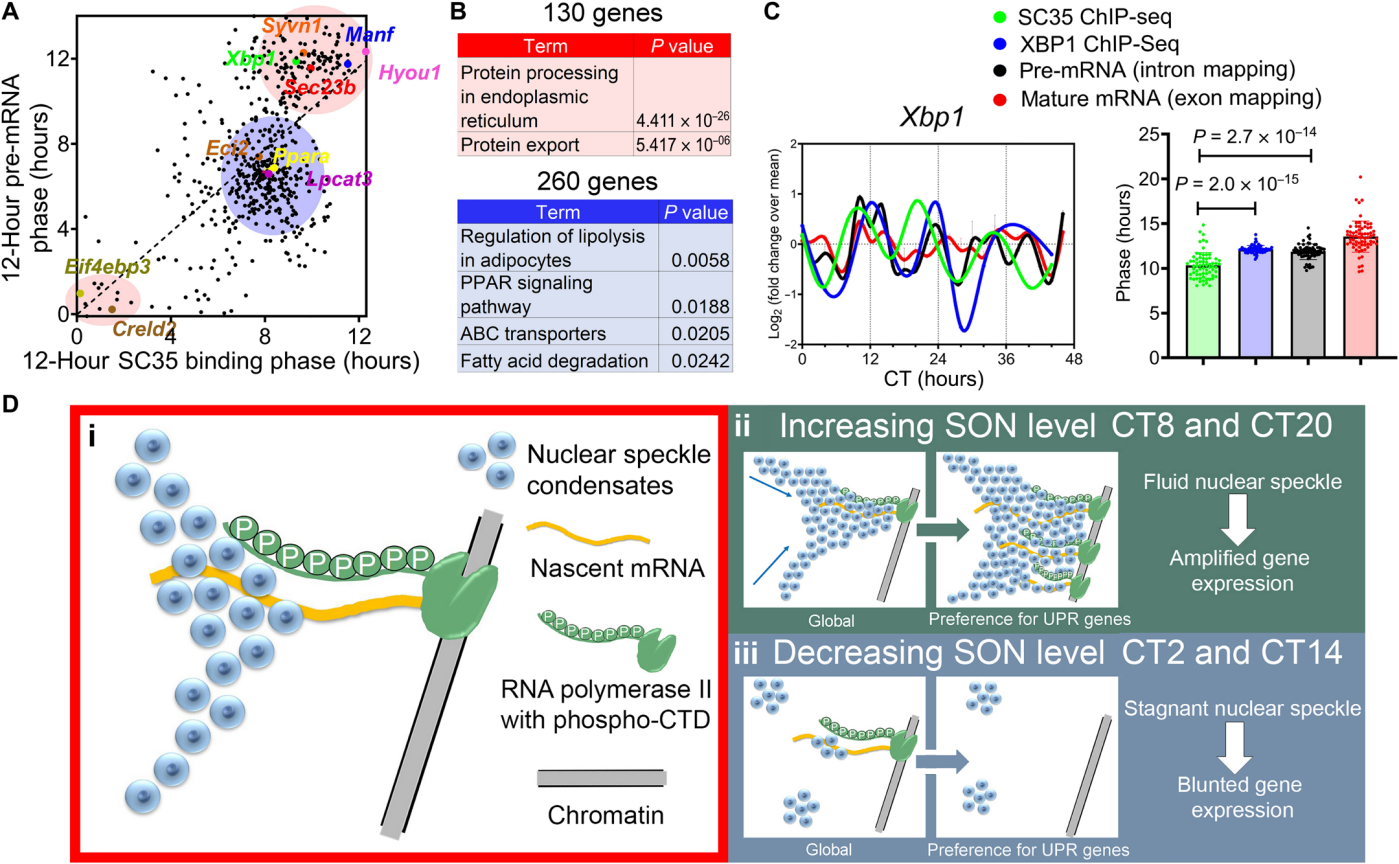
Proteostasis gene expressions are hypersensitive to nuclear speckle LLPS dynamic change. (**A**) Scatter plot demonstrating the phases of 12-hour rhythm of nuclear speckle–chromatin interactions (*x* axis) versus those of 12-hour rhythm of pre-mRNA expression (*y* axis) for 528 genes. Two major clusters of genes are highlighted in red and blue, respectively. (**B**) GO analysis of 130 genes highlighted in red and 260 genes highlighted in blue. (**C**) Quantification of the phases of 12-hour rhythms of nuclear speckle–chromatin interaction, XBP1s chromatin binding, and pre-MRNA and mature-mRNA expression of 69 genes that exhibit all four, with temporal profile for *Xbp1* gene also shown. Data are means ± SEM. (**D**) A simplified model demonstrating that SON can positively regulate nuclear speckle fluidity and their interactions with chromatin, and subsequent gene expression involved in proteostasis and UPR.

Focusing on 528 genes that exhibit very robust 12-hour rhythms of nuclear speckle–chromatin interactions (FDR = 0.2 among the 5365 genes with strong SC35 binding peaks) and XBP1s-dependent 12-hour rhythms of gene expression (FDR = 0.2 at both the pre-mRNA and mature-mRNA level) (fig. S11, A to D), we identified two major groups of genes with different phase relationship between the two. For 260 genes enriched in lipid metabolism and peroxisome proliferator–activated receptor (PPAR) signaling (blue area in Fig. 4, A and B), nuclear speckle–chromatin interaction peaks at CT8, closely following the peak of nascent mRNA expression at CT6 and matches the peak of mature-mRNA expression (fig. S11, E and F). For the majority of 130 genes enriched in ER stress and protein sorting pathways (red area in Fig. 4, A and B), the peaking times of the nuclear speckle–chromatin interactions precede those of nascent mRNA expression (above the diagonal line in Fig. 4A), which include genes like *Manf*, *Hyou1*, and *Sec23b* (Fig. 4A). For 69 genes with additional 12-hour XBP1s chromatin recruitment (*6*), the acrophase of nuclear speckle–chromatin interaction can even precede that of XBP1s chromatin binding, with *Xbp1* itself as a good example (Fig. 4C and fig. S11, G and H).

These results imply the existence of multitiered mechanisms for regulating nuclear speckle–chromatin interactions. As recently demonstrated, nuclear speckle condensates are thought to be “passively” recruited to chromatin following nascent mRNA transcription, mediated by RNA polymerase II hyperphosphorylation during transcriptional elongation (Fig. 4Di) (*26*). This mechanism is likely responsible for maintaining the core circadian clock and lipid metabolism gene expression, as the dynamics of their nuclear speckle–chromatin interactions closely follow their temporal gene expression change. The emerging data further suggest an additional layer of nuclear speckle–chromatin interaction control. Rather than responding to the transcriptional state of individual genes, nuclear speckle–chromatin interactions can also be modulated by the SON-mediated nuclear speckle LLPS dynamics on a global scale (Fig. 4D, ii and iii). In this case, the dynamics of nuclear speckle–chromatin interactions can even precede the subsequent gene expression change. These data further suggest that this second mode of “proactive” nuclear speckle–chromatin interaction is strongly implicated in the transcription regulation of proteostasis and UPR genes.

### SON transcriptionally amplifies the UPR and protects against proteome stress

**Fig. 5.**
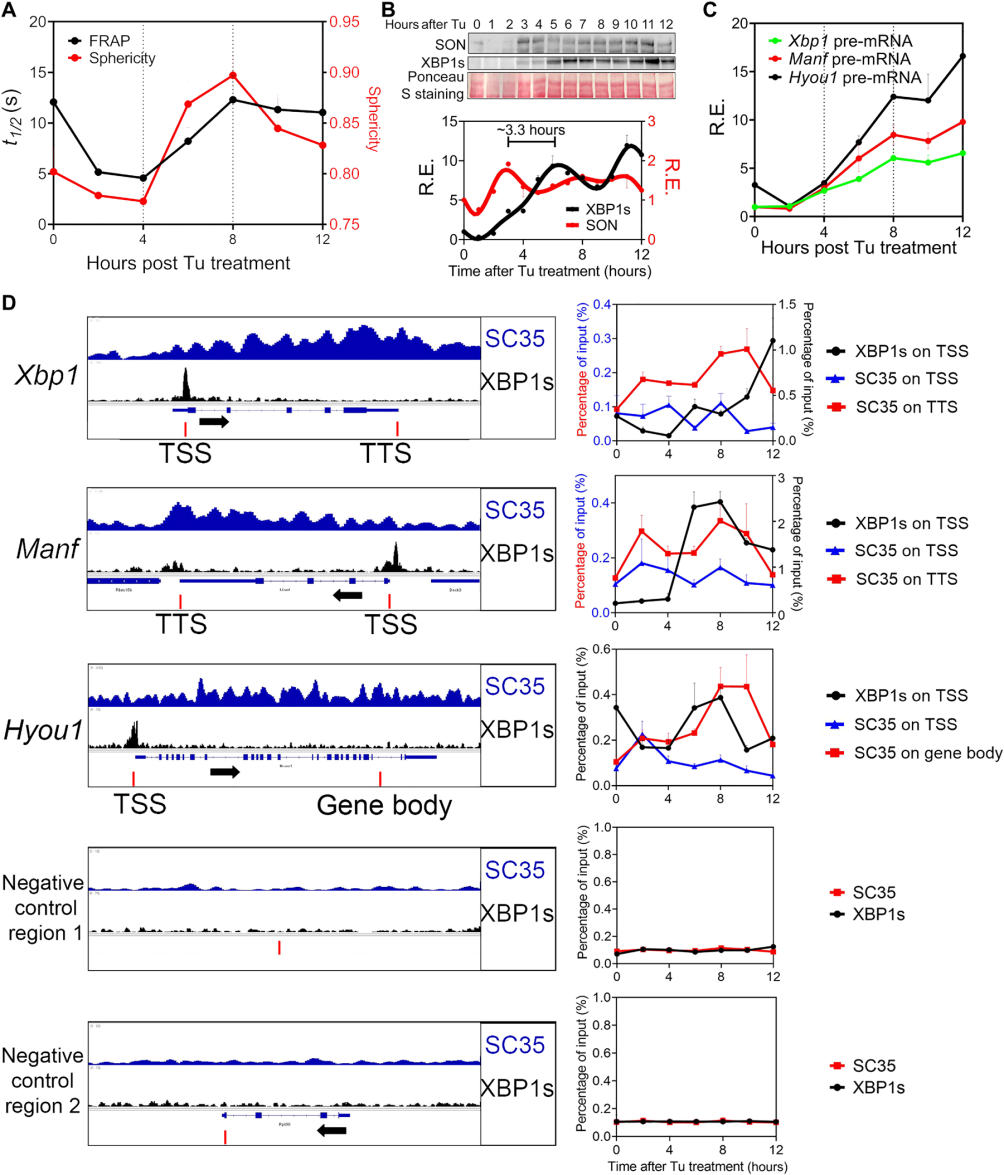
Temporal kinetics of UPR in response to low level of ER stress recapitulates endogenous 12-hour oscillator. MEFs were treated with Tu (100 ng/ml) for different hours. (**A**) FRAP assay and quantification of nuclear speckle sphericity. (**B**) Western blot and quantification (normalized to total Ponceau S staining intensity) of SON and XBP1s expression. (**C**) qPCR analysis of pre-mRNA level of different UPR genes. (**D**) Selected genes aligned for SC35 and XBP1s ChIP-seq signal from CT12 in XBP1*^Flox^* mice (left) and ChIP-qPCR of XBP1s and SC35 on selected regions (indicated by red bars) (right). Data are means ± SEM.

Before the discovery of the mammalian 12-hour oscillator, UPR was classically studied as a transient response to an insult to the ER (also known as ER stress). Transmitting a cascade of signals from ER to the nucleus, UPR ultimately activates three main transcription factors: XBP1s, activating transcription factor 4 (ATF4), and ATF6 (*31*). To seek more support for the causal role of SON and nuclear speckle LLPS on UPR gene regulation, we further examined the temporal kinetics of canonical UPR in response to a very low dose of ER stress inducer tunicamycin (Tu) (100 ng/ml) in MEFs. We noted that starting from 2 hours after Tu treatment, an immediate early increase of SON expression was concomitant with increasing nuclear speckle fluidity and diffuseness and increasing nuclear speckle–chromatin interactions in the gene bodies and/or TTSs of *Xbp1*, *Manf*, and *Hyou1* genes (Fig. 5, A to D). This immediate early SON-mediated nuclear speckle LLPS dynamics change precedes the increase in XBP1s promoter recruitment as well as *Xbp1*, *Manf*, and *Hyou1* gene expression with a phase advance of ~3.3 hours (Fig. 5, B to D), similar to what was observed in mouse liver in vivo (fig. S6E). We further observed a second wave of nuclear speckle–chromatin interactions peaking at 8 hours after Tu treatment that follows XBP1s promoter recruitment (Fig. 5D). This second wave is not associated with increased nuclear speckle fluidity and thus reflects “passive” chromatin recruitment of nuclear speckles during transcription elongation (Fig. 5A). This reduced nuclear speckle fluidity during the second wave is likely a result of a more stable interaction between nuclear speckle and chromatin during mRNA processing.

**Fig. 6.**
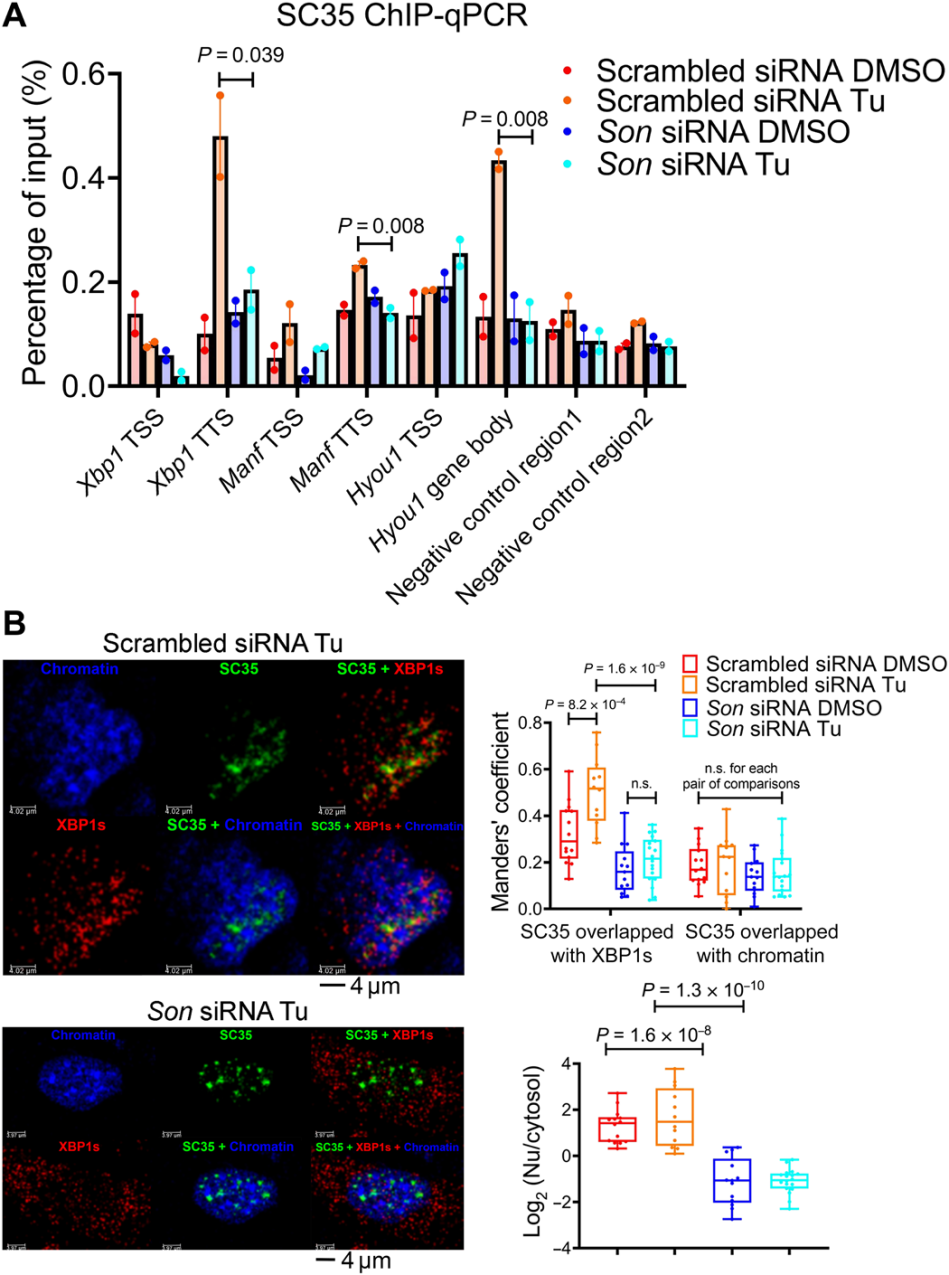
SON is required for colocalization of nuclear speckles with XBP1s during ER stress. MEFs were transfected with control or *Son* siRNAs and treated with Tu (100 ng/ml) for 6 hours. (**A**) ChIP-qPCR of SC35 on TSS and TTS of gene bodies of selective UPR genes. Data are means ± SEM. (**B**) Immunofluorescence of anti-XBP1s (red), GFP signal (green) from GFP::SC35 fusion protein and DAPI nuclei staining (blue), as well as merged images of either two or all three channels. Representative images (left), Manders’ coefficient quantification of colocalization of SC35/XBP1s and SC35/chromatin signals (top right), and quantification of log_2_-transformed ratio of nuclear to cytosol level of XBP1s (bottom right). Box and whiskers plot showing minimum to maximum values. n.s., not significant.

**Fig. 7.**
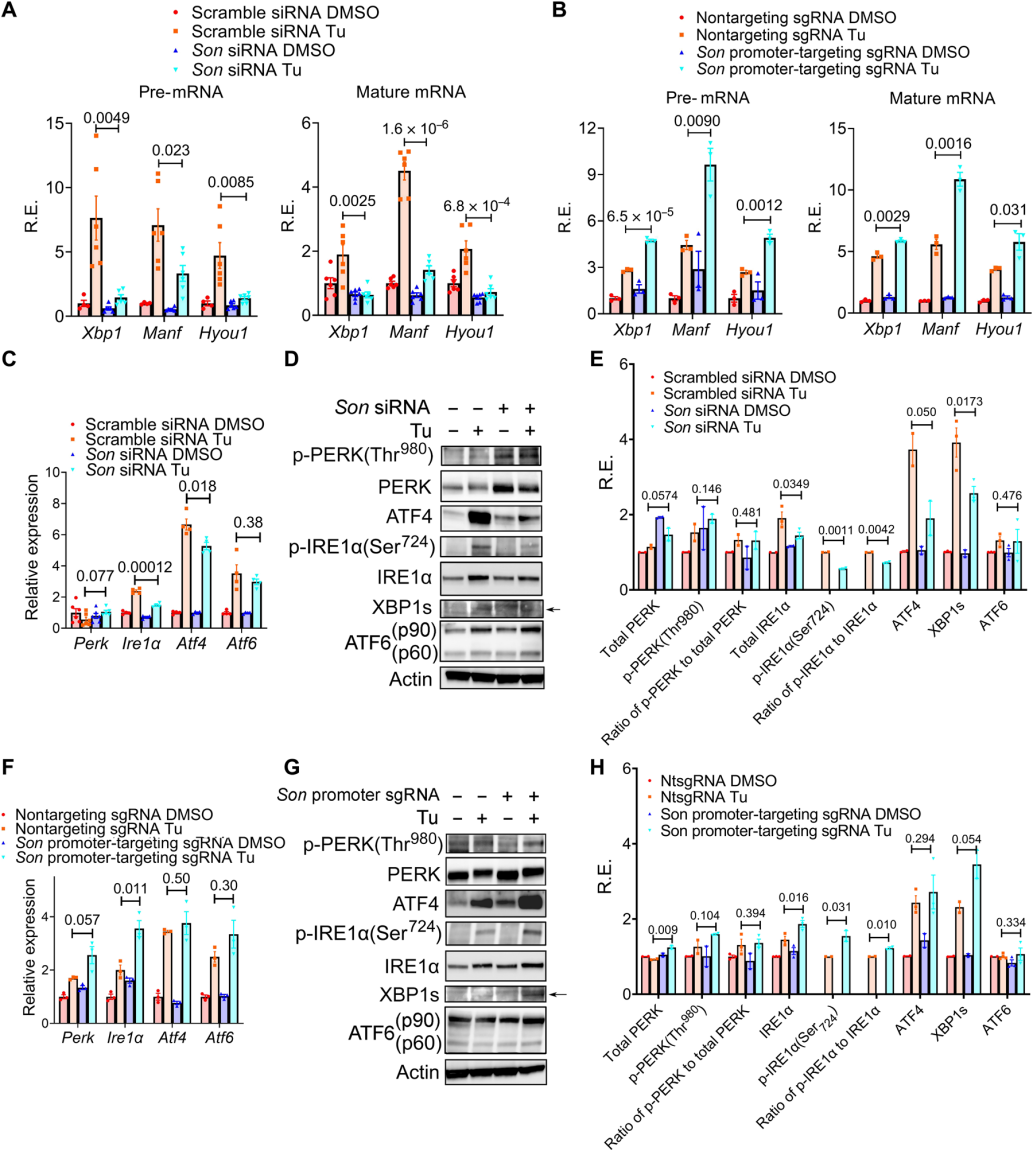
SON amplifies the UPR transcriptionally. (**A**) MEFs were transiently transfected with scramble control or *Son* siRNA and treated with Tu (100 ng/ml) for 6 hours, and qPCR analysis was performed on selective genes. (**B**) Control MEFs or MEFs with dCAS9-VPR–mediated stable overexpression of *Son* were treated with Tu (100 ng/ml) for 6 hours, and qPCR analysis was performed on selective genes. (**C** to **E**) MEFs were transiently transfected with scramble control or *Son* siRNA and treated with Tu (100 ng/ml) for 6 hours and qPCR analysis (C) and representative Western blot images (D) and quantification (E). (**F** to **H**) Control MEFs or MEFs with dCAS9-VPR–mediated stable overexpression of *Son* were treated with Tu (100 ng/ml) for 6 hours and qPCR analysis (F) and representative Western blot images (G) and quantification (H). For p-IRE1α, DMSO condition expression is too low to be accurately quantified. Data are means ± SEM.

To confirm the ChIP-qPCR results, we performed immunofluorescence and observed a dose-dependent increase in colocalization of XBP1s with nuclear speckles in response to ER stress in MEFs (fig. S12, A to D). siRNA-mediated knocking down of SON greatly reduces ER stress–induced nuclear speckle–chromatin interactions (Fig. 6A), disrupts XBP1s and nuclear speckle colocalization (Fig. 6B), and subsequently greatly blunts Tu-induced UPR at both the pre-mRNA and mature-mRNA level, while not affecting core circadian clock genes expression (Fig. 7A and fig. S13, A and B). By contrast, both stable and transient dCAS9-VPR–mediated overexpression of endogenous SON substantially amplifies UPR at the transcriptional level (Fig. 7B and fig. S13, C to F). UPR genes under SON control include not only ER stress–responsive output genes such as *Manf* and *Hyou1* but also core regulatory genes in the XBP1 and ATF4 branches of the UPR: *Xbp1* itself, *Ire1*α, and *Atf4* (Fig. 7, C to H), the latter of which appears to respond to a narrower range of SON level, as overexpression of SON has little effect on *Atf4* gene expression (Fig. 7F). By contrast, SON does not significantly affect *Atf6* expression (Fig. 7, C and F). The dichotomy of the effects of SON on *Atf4* and *Atf6* gene expression during transient ER stress in MEFs is consistent with their differential temporal gene expression profiles in mouse liver in vivo: While both *Atf4* and *Atf6* pre-mRNA exhibit 12-hour rhythms in wild-type mouse liver, only the former exhibits a robust 12-hour rhythm of XBP1s-dependent nuclear speckle–chromatin interaction and dampened 12-hour rhythm of expression in XBP1 *^LKO^* mice (fig. S13, G to J). These UPR gene expression changes in response to SON manipulation are similarly conserved at the protein level (Fig. 7, D, E, G, and H) and largely recapitulated with a different ER stress inducer, dithiothreitol (DTT) (fig. S14, A and B). Last, we ruled out the possibility that protein synthesis is involved in the transcriptional regulation of the early stage of UPR by SON, as neither changes of protein synthesis rate (fig. S15, A and B) nor the relative amount of phospho-PERK (protein kinase R-like endoplasmic reticulum kinase) (Thr^980^) levels (Fig. 7, D, E, G, and H) was observed under SON knocking down or overexpression conditions. Together, our data strongly indicate that SON can rapidly amplify XBP1s and ATF4 (although to a lesser extent)–mediated UPR, while having very modest effects on the ATF6 branch at the transcriptional level.

**Fig. 8.**
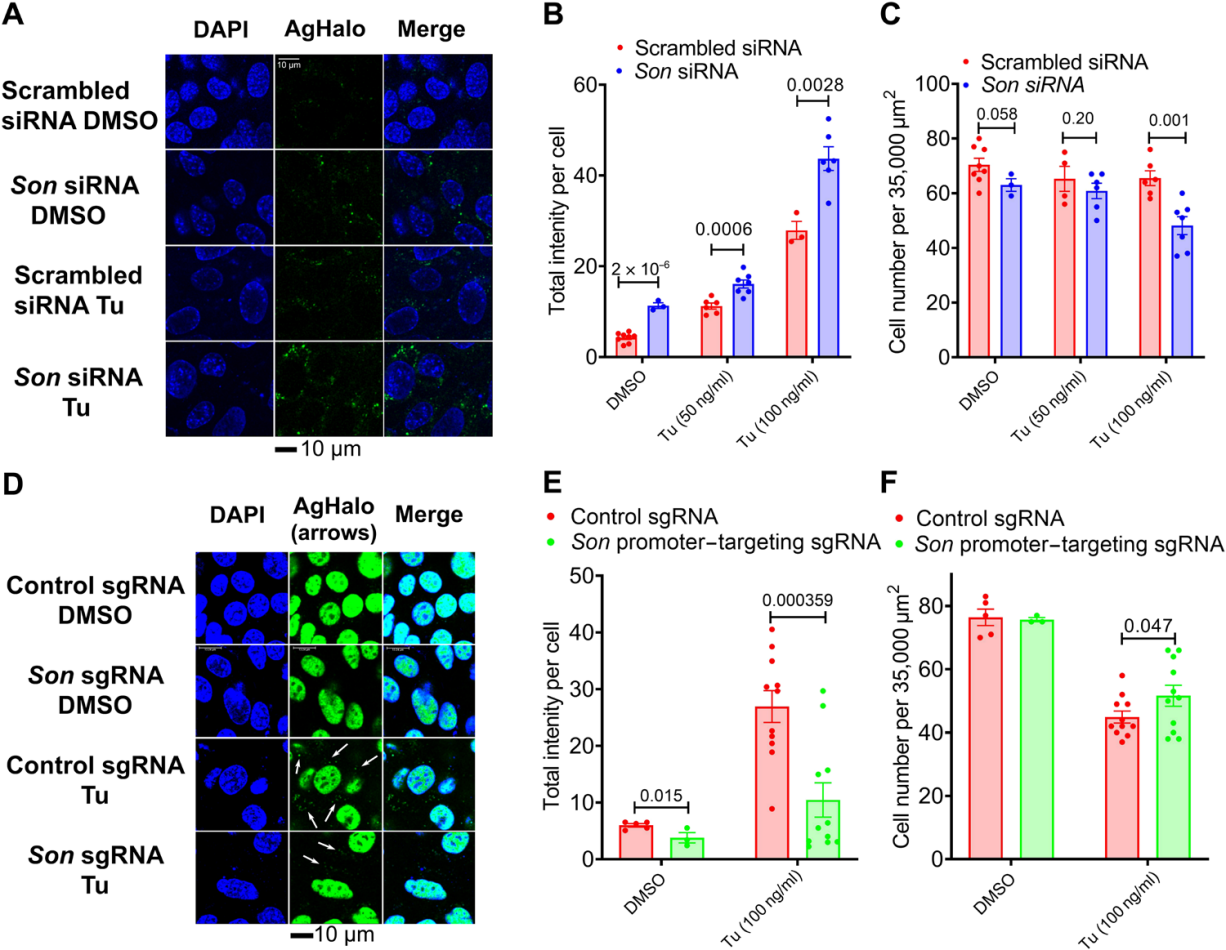
SON protects cells against proteome stress. (**A** to **C**) MEFs were transiently transfected with AgHalo_ER_ plasmid and then further transfected with control or *Son* siRNAs and treated with different concentrations of Tu for 16 hours before being subjected to confocal live imaging. Representative confocal images of DAPI staining and AgHalo_ER_-probe conjugates in response to Tu (C). Quantification of averaged AgHalo-probe intensity per cell (**D**) and averaged cell number per area of 35,000 μm^2^ (**E**). Each data point in (B) is averaged intensity per cell calculated from each image of 35,000 μm^2^ area. (D to **F**) dCAS9-VPR GFP::SC35 MEFs expressing control or *Son* promoter–targeting sgRNA were transiently transfected with AgHalo_ER_ plasmid and then treated with tunicamycin (Tu; 100 ng/ml) for 16 hours before subject to confocal live imaging. Representative confocal images of DAPI staining and ER-targeting AgHalo_ER_-probe conjugates in response to Tu (100 ng/ml) (D). White arrows indicate ER-targeting AgHalo_ER_-probe conjugates. Quantification of averaged AgHalo-probe intensity (nonnuclear portion) per cell (E) and averaged cell number per area of 35,000 μm^2^ (F). Each data point in (E is averaged intensity per cell calculated from each image of 35,000 μm^2^ area. Data are means ± SEM.

To determine the functional importance of SON in regulating proteostasis in the ER, we went on to detect and quantify ER proteome stress in response to SON manipulation in MEFs. We used a previously published Halo-tag mutant (K73T/L172Q) prone to ER-localized aggregation (AgHalo_ER_) (*32*). The AgHalo_ER_ sensor was labeled with solvatochromic fluorogenic probe (P1), which turns on fluorescence only upon its misfolding and aggregation (*33*). As expected, live cell imaging of MEFs expressing AgHalo_ER_ labeled by P1 probe demonstrated that the AgHalo_ER_ was well folded with little green fluorescence signal under basal conditions and formed granular green fluorescent structures after 16-hour Tu treatment (Fig. 8A). Under both basal and Tu conditions, a notable increase in AgHalo_ER_ staining intensity was observed in SON-depleted MEFs (Fig. 8, A and B), which is concomitant with reduced cell number in response to a higher concentration of Tu treatment under SON-depleted condition (Fig. 8C). Conversely, dCAS9-VPR–mediated SON overexpression significantly reduced AgHalo_ER_ misfolding and protected against ER stress–induced cell death (Fig. 8, D to F). Collectively, these data indicate that by amplifying the UPR, SON can protect cells against proteome stress.

Consistent with its role in amplifying UPR during transient ER stress, *Son* knockdown further dampened the 12-hour oscillation of *Manf* promoter–driven luciferase activity, while having no apparent effects on circadian *Bmal1* promoter–driven luciferase rhythm (fig. S16, A to F). Together, these results strongly support a positive causal role of SON and nuclear speckle LLPS on the transcriptional regulation of proteostasis, both for UPR in response to a transient ER stress and the cell-autonomous 12-hour oscillator.

### Correlative SON and UPR gene expression dynamics across mouse life span

**Fig. 9.**
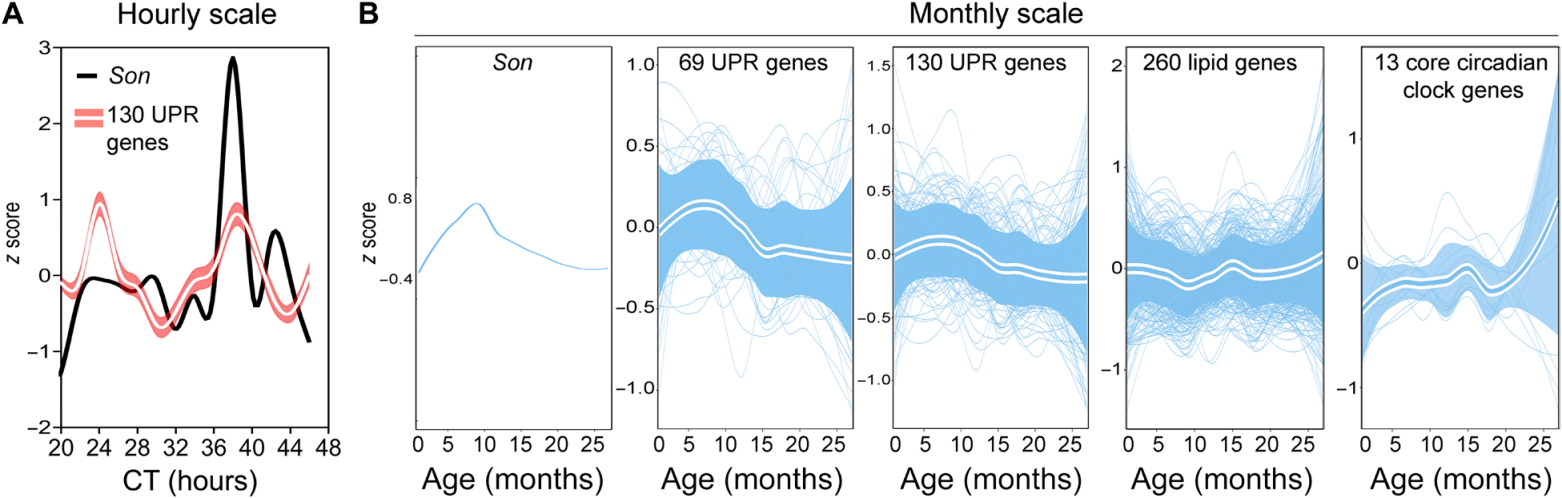
Correlative hepatic *Son* and UPR gene expression dynamics is observed across the mouse life span. Expression of *Son* and UPR genes in a 28-hour time window (**A**) or across the entire mouse life span (**B**) in the mouse liver. Solid line, mean; shaded area, 95% confidence interval.

To determine whether the hourly *Son* and UPR gene expression dynamics can be extrapolated to a longer temporal scale, we analyzed a recently published RNA-seq data in multiple mouse tissues across a 27-month life span (*34*). We observed strong correlative *Son* and UPR mRNA expression dynamics in the liver, skin, heart, pancreas, and bone across the entire mouse life span (Fig. 9, A and B, and fig. S17, A to D). In addition to the liver, 12-hour rhythms of *Son* and UPR mRNA were also observed in mouse skin (*35*), heart (*36*), and pancreas (*37*) (fig. S17, A to C), with the latter exhibiting a very intriguing fractal feature of antiphase oscillations of *Son* and UPR gene expression at the periods of 12 hours and ~16 months (fig. S17C). The anticorrelation between *Son* and UPR mRNA in mouse pancreas could be due to a large discordance between the mRNA and protein level of SON and warrants further investigation. In contrast to UPR genes, no strong correlation was observed between the gene expression dynamics of *Son*, and lipid and core circadian clock genes in different tissues across the mouse life span (Fig. 9, A and B, and fig. S17, A to D).

## DISCUSSIONS

**Fig. 10.**
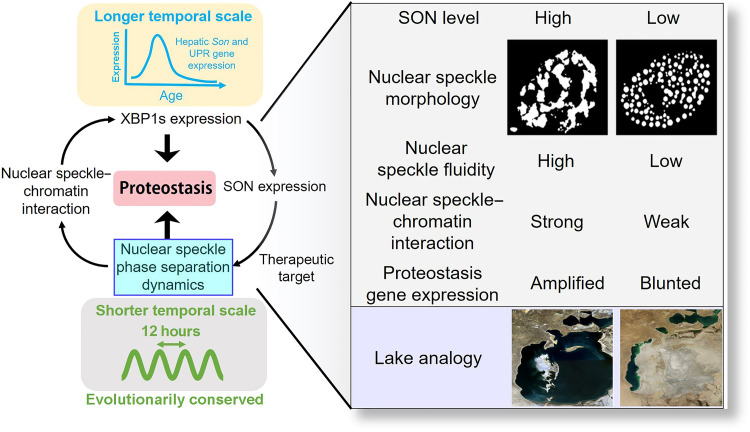
XBP1s-SON axis spatiotemporally controls nuclear speckle LLPS to regulate proteostasis. An analogy would be the water level fluctuations in a lake: Nuclear speckles with higher SON level is like a lake filled with free-flowing water with fast dynamics, while nuclear speckles with reduced SON expression resemble a nearly dried-out lake with a few disconnected pools of stagnant water. Our study further indicates that the nuclear speckle LLPS may be a previously unidentified therapeutic target for pathologies that arise as a result of dysregulated proteostasis. Please refer to Discussions for details.

While considerable progress has been made toward understanding the biophysical properties and the biological functions of biomolecular condensates and LLPS (*38*), it remains elusive whether LLPS dynamics are under the control of “autonomous clocks” (*39*). Our study hereby demonstrates the existence of an evolutionarily conserved XBP1s-SON axis that integrates the 12-hour oscillator with nuclear speckle LLPS to spatiotemporally program proteostasis (Fig. 10). As the scaffolding protein of nuclear speckle condensates (*19*), SON level can dictate the mechanisms by which nuclear speckles phase separate. Nuclear speckles with a lower concentration of SON exhibit features reminiscent of punctate nucleation and growth, while those with higher SON expressions exhibit early-stage coarsening morphologies—connected network-like condensates—associated with spinodal decomposition (*15*). While the coarsening of condensates will eventually result in the coalescence of all droplets into a single large sphere in an ideal situation driven by surface tension, the chromatin likely greatly slows down this process so that an intermediate connected network-like nuclear speckle morphology can occur (*40*). Both the phase and amplitude of SON oscillation further align with the prediction by the phase separation diagram. More fascinatingly, nuclear speckles with lower SON expression are also more stagnant, whereas much more fluid dynamics are found in nuclear speckles with higher SON expression. Whether the dynamics of nuclear speckle LLPS is intrinsic to the way by which LLPS occurs (nucleation or spinodal decomposition) remains to be determined. One possibility is that nucleation-mediated LLPS will result in many individually isolated condensates, and the barriers of liquid droplets would limit molecular diffusion between dense and dilute phases, while during spinodal decomposition, the connected network-like condensate morphology would greatly favor rapid molecular diffusion within the condensates. The latter would also facilitate the rapid delivery of splicing factors to transcription foci during ER stress to amplify the UPR at the cotranscriptional splicing/transcription elongation stage (*18*). Conversely, lower SON expression–associated stagnant nuclear speckles are sequestered away from chromatin and thereby would greatly dampen the UPR (Figs. 4D and 10). It is worth noting that besides SON, we are not ruling out the possibility that other nuclear speckle proteins and/or RNAs may also regulate the 12-hour nuclear speckle LLPS dynamics, with the Pickering agent, which has recently been found to be regulating P granules coarsening, being a tantalizing candidate (*41*). Our results further reconcile the debate on the exact roles of nuclear speckles in gene regulation: whether the nuclear speckles mainly function as the “storage facility” of mRNA processing factors away from chromatin, or they can actively participate in gene regulation process via physical engagement with chromatin (*42*). We showed here that nuclear speckles can function as both, but these two functions are probably temporally separated because of the oscillation of SON expression and the according changes in their LLPS dynamics and propensity to associate with chromatin.

Thus, what could be the biological advantages of having a 12-hour rhythm of nuclear speckle LLPS dynamics? Because the acrophases (the time period in a cycle during which the cycle peaks) of 12-hour rhythms of gene expression are strongly biased toward dawn and dusk, we previously proposed a vehicle-cargo hypothesis to elucidate the distinct functions of 12-hour versus the circadian rhythms (*6*). We argued that the 12-hour rhythm accommodates increased demands for gene expression/processing at the two biological “rush hours” (dawn and dusk) by elevating the global traffic capacity of the CEDIF. This connects and tunes rates of mRNA, protein, and lipid metabolism to the 12-hour cycle of metabolic stress (thus acting as the vehicle). The circadian clock, on the other hand, dictates the particular genes/gene products processed at each rush hour (thus acting as the cargo). We posit that having increased nuclear speckle fluidity at early morning/early afternoon enables the in-land animals to anticipate and, subsequently, to rapidly turn on UPR genes to cope with heightened metabolic stress associated with transition periods later at dawn and dusk. This feature is likely co-opted from the circatidal clock of marine animals, who probably used a similar system to adapt to the 12-hour environmental cues resulting from tidal changes. The hypersensitivity of proteostasis gene expression to nuclear speckle LLPS dynamics would ensure tightly coupled mRNA and protein metabolic processes, which, in turn, can entail a highly efficient genetic information transfer across multiple compartments within the cell. The fact that manipulating the LLPS dynamics of nuclear speckle is sufficient to alter the transcriptional output of proteostasis genes further suggests that the nuclear speckle LLPS may represent a previously unidentified (chrono)-therapeutic target for pathologies associated with dysregulated proteostasis (Fig. 10). A recent study found that the nuclear speckle can also amplify p53-mediated gene expression (*43*). Because p53 is known to be part of the DNA damage response, it is reasonable to conjecture that boosting nuclear speckle function via modulating its LLPS may exert beneficial effects via simultaneously augmenting multiple adaptive stress responses, thereby potentially enhancing the overall antiaging hormesis (*44*).

On a more philosophical note, our study is a good example of how seemingly unrelated biological processes can be tightly connected through the time dimension, in this case, their 12 hours of rhythmicity. In this study, we showed that one can use frequency spectrum similarity to interrogate genetic interactions within a cell. We hope that our study will make the scientific community think more deeply about the temporal dimension of their biological problems and facilitate the achievement of the ultimate goal of “mapping the four-dimensional atlas” of biological processes.

## MATERIALS AND METHODS

### Animals

XBP1*^Flox^* mice were as previously described (*45*). XBP1*^LKO^* mice were generated by crossing albumin-CRE mice with XBP1^Flox^ mice. All mice are in C57BL/6 background, male, and between 3 and 4 months of age. Mice were first entrained under 12-hour light/12-hour dark conditions for 2 weeks before they were transferred to constant darkness for 24 hours. Mice were then euthanized at a 2-hour interval for a total of 48 hours. Mice were fed ad libitum during the entire experiment. The animal studies were carried out in accordance with the National Institutes of Health guidelines and were granted formal approval by the University of Pittsburgh’s Institutional Animal Care and Use Committee (approval numbers IS00013119 and IS00013119).

### Plasmids

Mouse nontargeting, *Bmal1* and *Xbp1* single-guide RNAs (sgRNAs) were cloned into the lentiCRISPRv2 plasmid as previously described (*46*), which is a gift from T. Finkel. The sgRNA sequences (in bold) are as follows: nontargeting control, caccg**AAATGTGAGATCAGAGTAAT**; *Bmal1*, caccg**CCCACAGTCAGATTGAAAAG**; and *Xbp1*, caccg**GGAGCAGCAAGTGGTGGATT**. dCAS9-VPR plasmid (*47*) was a gift from G. Church (Addgene, plasmid no. 63798; http://n2t.net/addgene:63798; RRID:Addgene_63798). Mouse nontargeting, *Son* promoter–targeting sgRNAs were cloned into the pLenti-SpBsmBI-sgRNA-Hygro plasmid (*47*), which was a gift from R. Maehr (Addgene, plasmid no. 62205; http://n2t.net/addgene:62205; RRID: Addgene_62205). The sgRNA sequences are as follows: nontargeting control, aaatgtgagatcagagtaat; *Son* promoter-targeting sgRNA1, atggcggccgagttcgtgcg; and *Son* promoter–targeting sgRNA2, taggagtccccgcaggctga. XBP1s overexpression plasmid PHAGE-Flag-XBP1s was as previously described (*4*).

### siRNA/sgRNA transient transfections

MEFs were transfected with 10 μM of different siRNAs for 24 to 48 hours with Lipofectamine RNAiMAX reagents (Life Technologies) per the manufacturer’s instructions. Sources of siRNA are as follows: siGENOME Non-Targeting siRNA pool (Dharmacon, D-001206-1305) and siGENOME SMARTpool SON siRNA (Dharmacon, L-059591-01-0005). For transient transfection of *Son* promoter–targeting sgRNAs, nontargeting sgRNA or *Son* promoter–targeting sgRNA1 and sgRNA2 were synthesized in vitro by the EnGen sgRNA Synthesis Kit, *S. pyogenes* per the manufacturer’s protocol. MEFs stably expressing dCAS9-VPR were transfected with 10 μM of different sgRNAs and cotreated with Tu (100 ng/ml) for 6 hours.

### Cell culture

MEFs were isolated from male SRC-2*^fl/fl^* mice and immortalized by SV40 T antigen as previously described (*48*). For dexamethasone (Dex) treatment, MEFs were cultured in Dulbecco’s modified Eagle’s medium (DMEM) (glucose, 4.5 g/liter) supplemented with 10% fetal bovine serum (FBS) and treated with 100 nM Dex for 30 min and then washed with 1× phosphate-buffered saline (PBS) before being cultured in the same medium. For serum shock, MEFs were cultured in DMEM (glucose, 4.5 g/liter) supplemented with 10% FBS and treated with 50% horse serum for 2 hours and then washed with 1× PBS before being cultured in the same medium. For Tu treatment, MEFs were treated with Tu (100 ng/ml) [in dimethyl sulfoxide (DMSO)] for different times, unless it is otherwise indicated. For all cell culture experiments, cells were cultured at 37°C with 5% CO_2_.

### Establishing stable cell line

For sgRNA-mediated Bmal1/XBP1 knockout MEFs, lentiviruses packaged in human embryonic kidney (HEK) 293T cells with cotransfection of lentiCRISPRv2, pMD2.G, and psPAX2 plasmids were used to infect MEFs with a multiplicity of infection of 3. Stable MEFs were selected in the presence of puromycin (4 μg/ml). For SON trans-activation MEFs, MEFs were first transfected with dCAS9-VPR plasmid, and those stably expressing dCAS9-VPR were selected in the presence of G418 (200 μg/ml). dCAS9-VPR stably expressing MEFs were further infected with lentiviruses packaged from HEK293T cells transfected with pLenti-SpBsmBI-sgRNA-Hygro, pMD2.G, and psPAX2 plasmids. Final SON trans-activation MEFs (with *Son* promoter–targeting sgRNA1) were selected in the presence of G418 (200 μg/ml) and hygromycin (200 μg/ml).

### Generation of GFP::SC35 cells

The CRIS-PITCh (v2) system as described in (*49*) was used to generate an GFP::SC35 knock-in cell line. The CRIS-PITCh (v2) system requires an “all-in-one” expressing CAS9, a CRISPR guide strand targeting a cut site in the desired genomic locus, and a CRISPR guide strand targeting the CRIS-PITCh (v2). The CRIS-PITCh (v2) contains a sequence designed to recombine into the desired locus and insert a sequence [Puro–T2A–enhanced GFP (EGFP)]. All-in-one vector: Oligos (Thermo Fisher Scientific) (forward) 5′-CACCTGTCCGGGGCGTTAGGGTCT-3′ and (reverse) 5′-AAACAGACCCTAACGCCCCGGACA-′3 were annealed using Nuclease Free Duplex Buffer (Integrated DNA Technologies 11-01-03-01). The plasmid pX330_1x2_addgene-58766-sequence-202009 (Addgene, www.addgene.org/58766/) was digested with BbsI-HF [New England Biolabs (NEB), R3539S] (Antarctic Phosphatase M0289S) and gel purified. The resulting fragment was ligated to the annealed oligos using T4 ligase (M0202S). The Pitch guide RNA was cleaved form the Pitch_Cas9_addgene-63670-pX330S-2-Cas9PITCh-106070 (Addgene, www.addgene.org/browse/article/16395/) plasmid using BsaI-HFv2 (NEB, R3733S). This fragment was gel purified and ligated to BsaI BsaI-HFv2 (NEB, R3733S) cleaved and purified SRSF2 guide RNA vector. The resulting vector contained sequences to express the SRSF2 locus-specific guide RNA and the Pitch guide RNA. CRIS-PITCh (v2): The Puro-T2A-EGFP region was amplified from the mp132-psicor-puro-t2a-egfp plasmid [ViraCore at the University of California, San Francisco (UCSF), viracore.ucsf.edu] using the following primers (Thermo Fisher Scientific): Q5 High-Fidelity 2X Master Mix (NEB, M0492S) (forward) 5′-CCGCGTTACATAGCATCGTACGCGTACGTGTTTGGTGTCCGGGGCGTTAGGGTCTATGACCGAGTACAAGCCC-3′ and (reverse) 5′-CAGCATTCTAGAGCATCGTACGCGTACGTGTTTGGGGCGGGCGGCCGTAGCTCATGGATCCGGGCTTGTACAGCTCGTCCATG-3′. The resulting PCR fragment was gel purified and joined to the Mlu I (NEB, R0198S) digested pCRIS-PITChv2-FBL (www.addgene.org/63672/) using Gibson Assembly (NEB, R3539S). The all-in-one vector (1.2 μg) and the CRIS-PITCh (v2) (0.6 μg) were transfected into a 100-mm dish of low-passage number MEFs using Lipofectamine 3000 (Invitrogen, 100022052) and P3000TM Reagent (Invitrogen, 100022058) and Optimem (Gibco, 31985-070). The transfected MEFs were cultured [DMEM (Gibco, 21013-024), 10% FBS (HyClone, SH30910.03), 1% penicillin-streptomycin (Gibco, 15140-122), 1% sodium pyruvate (Gibco, 11360-070)] in increasing concentrations [puromycin, 2 to 5 μg/ml (Gibco, A11138-03)] for selection. Colonies were diluted using 15-cm cell culture plates. Colonies were examined for EGFP fluorescence and were subsequently cloned using cloning circles (Sigma-Aldrich, Z370789) and trypsin EDTA (Gibco, 25200072).

### Real-time luminescence assay

Stable *Manf*-dluc (*6*) or *Bmal1*-dluc MEFs (*6*) were cultured in DMEM (glucose, 4.5 g/liter) supplemented with 10% FBS and treated with 50% horse serum in DMEM for 2 hours or 100 nM for 30 min before being subjected to real-time luminescence assay using a LumiCycle (Actimetrics) as previously described (*6*). Briefly, after serum shock treatment, MEFs were washed with 1× PBS and cultured with DMEM (glucose, 4.5 g/liter) supplemented with 0.1 mM luciferin and 10 mM Hepes buffer in 35-mm tissue culture dishes in the absence of serum and transferred immediately to LumiCycle for real-time luminescence analysis. Periods of oscillation were identified by embedded Periodogram function. For siRNA-treated MEFs, MEFs were transfected with nontargeting or *Son* siRNA for 48 hours before being subjected to serum shock and real-time luminescence assay as described above.

### Immunofluorescence

Immunofluorescence was performed as previously described (*50*). Briefly, the liver Optimal cutting temperature (OCT) sections or cells cultured in chamber slide were fixed in cold acetone for 10 min at −20°C. The sections were then air dried, rehydrated with PBS, and permeabilized with PBS + 0.1% Triton X-100. The sections were then blocked with 10% goat serum at room temperature for 1 hour. Primary antibodies against SC35 (Abcam, ab11826), XBP1s (BioLegend, 658802), and SON (Abcam, 121033) were conjugated to Alexa Fluor 488 or Alexa Fluor 555, respectively, per the manufacturer’s protocol and added to the OCT section at 1:1000 dilution overnight at 4°C. Next day, sections were washed five times with PBS and counterstained with 4′,6-diamidino-2-phenylindole (DAPI) before mounting (with ProLong Gold Glass) and imaging using Leica SP8 lightening confocal microscope (Leica Microsystems). Three-dimensional construction from *z*-stack images was performed using Volume Viewer from ImageJ.

### Image analysis

All image analyses were performed in Cell Profiler (version 3.1.5). Quantification of the shape of nuclear speckles was performed with customarily written pipelines in Cell Profiler. For speckle *i*, the sphericity is defined as in Eq. 1

$$\text{Sphericity}\;i=2\sqrt{\pi }*\sqrt{\text{area}\;}i÷\text{circumference}\;i$$ (1)

so that a perfect circle will have a sphericity of 1, and a line will have a sphericity of 0. To calculate the average sphericity of a single cell that have *k* speckles, we calculated the area-weighted average as described in Eq. 2

$$\text{Average sphericity}/\text{cell}=\sum_{1}^{k}\text{Sphericity}\;i\;\times \;\text{area}\;i/\sum_{1}^{k}\text{area}\;i$$ (2)

### Time-lapse microscopy

Time-lapse imaging was performed using SP8 lightening confocal microscope (Leica Microsystems) with Okolab stage top incubator to maintain constant CO_2_ (5%), temperature (37°C), and humidity (90%). Cells were plated into an eight-well chamber slide in full DMEM, and images were taken every 15 min using autofocus function. For imaging of cells in multiple wells simultaneously, Mark and Find feature was used to ensure accurate capture of the same cells.

### Fluorescence recovery after photobleaching

FRAP was performed using a Leica SP8 lightening confocal microscope (Leica Microsystems) with 488-nm laser. Bleaching was performed using 100% laser power with 3.6 ms per pixel dwell time for five cycles, and images were collected every 1.29 s for 50 frames after bleaching. Fluorescence intensity at the bleached spot, a control unbleached spot, and background was measured using the embedded FRAP Profiler. Background intensity was subtracted, and values are reported relative to the unbleached spot to control for photobleaching during image acquisition. The recovery half-life (*t*_1/2_) was calculated by the online easyFRAP tool (https://easyfrap.vmnet.upatras.gr/) (*51*) by fitting to single or double exponential equations with the better fit (larger *R*-square values). For temporal FRAP analysis, different cells were selected for FRAP at each time point after Tu treatment or serum synchronization to minimize phototoxicity to cells due to repeated photobleaching.

### ER proteostasis assay

MEFs were seeded in an eight-well chamber slide and transiently transfected with plasmid expressing cytomegalovirus (CMV) promoter–driven ER-localized aggregation-prone Halo-tag mutant (K73T/L172Q) (AgHalo_ER_) (*32*). After 24 hours, the same cells were transfected with scrambled or *Son* siRNA for another 24 hours. Then, cells were treated with DMSO vehicle control or Tu (50 ng/ml or 100 ng/ml) for 16 hours. After that, cells were replaced with fresh DMEM containing 1 μM P1 to label AgHalo_ER_ protein for 30 min and then costained with Hoechst 33342/DAPI for nuclei. dCAS9-VPR GFP::SC35 MEFs expressing control or *Son* promoter–targeting sgRNA were transiently transfected with AgHalo_ER_ plasmid and then treated with Tu (100 ng/ml) for 16 hours before being subjected to confocal live imaging. Confocal images were obtained using a Leica SP8 confocal microscope (Leica Microsystems). The P1 signal was visualized with a blue argon laser (488 nm), and the Hoechst/DAPI signal was visualized using an ultraviolet laser (405 nm). Quantification of intensity was performed with Cell Profiler (version 3.15). For GFP::SC35 MEFs, only green signals that do not overlap with Hoechst staining (nucleus) were quantified.

### Quantification of protein synthesis rate

The Click-iT HPG Alexa Fluor 594 protein synthesis HCS kit (Thermo Fisher Scientific) was used to measure protein synthesis in vitro. Briefly, MEFs cultured in chamber slides were treated with DMSO or Tu (100 ng/ml) for 5.5 hours before being pulsed with 50 μM methionine analog l-homopropargylglycine in methionine-free medium for 0.5 hours. Cells were fixed with 2% formaldehyde and permeabilized by 0.5% Triton X-100 and then underwent a ligation reaction for 30 min in the dark. Nuclei were further counterstained with Hoechst. Representative photomicrographs were obtained with a Leica SP8 confocal microscope (Leica Microsystems), and cell average intensity of Alexa Fluor 594 signal was measured using Cell Profiler software (version 3.15). Only cytosolic Alexa Fluor 594 signals (those not overlapped with nuclear Hoechst staining) were measured.

### Immunoblot

Nuclear extracts were made from liver according to previously published protocol (*52*). Protein concentrations were determined by Bradford assays (Bio-Rad), and aliquots were snap frozen in liquid nitrogen and stored at −80°C until usage. Immunoblot analyses were performed as described previously (*53*). Briefly, 25 μg of proteins separated by 4 to ~20% gradient SDS–polyacrylamide gel electrophoresis gels (Bio-Rad) were transferred to nitrocellulose membranes, blocked in TBST buffer supplemented with 5% bovine serum albumin or 5% fat-free milk, and incubated overnight with primary anti-SON antibody (Abcam, 121033), anti-BMAL1 antibody (Abcam, 3350), anti-PERK (Cell Signaling Technology, no. 3192), anti–phospho-PERK (Thr^908^) (Thermo Fisher Scientific, MA5-15033), anti-ATF4 (Cell Signaling Technology, no. 11815), anti-IRE1α (Cell Signaling Technology, no. 3294), anti–phospho-IRE1α (Ser^724^) (ABclonal, AP0878), anti-XBP1s (BioLegend, 658802), anti-ATF6 (Santa Cruz Biotechnology, sc-166659), and β-actin (Cell Signaling Technology, no. 12620) at 4°C. Blots were incubated with an appropriate secondary antibody coupled to horseradish peroxidase at room temperature for 1 hour and reacted with Enhanced chemiluminescence (ECL) reagents per the manufacturer’s (Thermo Fisher Scientific) suggestion and detected by the Bio-Rad ChemiDoc MP Imaging System.

### Quantitative reverse transcription polymerase chain reaction

Total mRNA was isolated from MEFs or mouse liver with a PureLink RNA mini kit (Life Technologies) with additional on-column deoxyribonuclease digestion step to remove genomic DNA per the manufacturer’s instructions. Reverse transcription was carried out using 5 μg of RNA using SuperScript III (Life Technologies) per the manufacturer’s instructions. For gene expression analyses, cDNA samples were diluted 1/30-fold (for all other genes except for 18*S* RNA) and 1/900-fold (for 18*S* RNA). qPCR was performed using the SYBR Green system with sequence-specific primers. All data were analyzed with 18*S* or β*-actin* as the endogenous control. qPCR primer sequences are as follows, and all primers span introns, except for primers for quantifying pre-mRNAs: mouse total *Xbp1*, (forward) gggtctgctgagtcc and (reverse) cagactcagaatctgaagagg; mouse total *Xbp1* pre-mRNA, (forward) GTTAAGAACACGCTTGGGAATG and (reverse) TGGAGGTCCAGAACACAAAC; mouse *Arntl*, (forward) gccccaccgacctactct and (reverse) tgtctgtgtccatactttcttgg; mouse *Per1*, (forward) tcctcctcctacactgcctct and (reverse) ttgctgacgacggatcttt; mouse *Per2*, (forward) caacacagacgacagcatca and (reverse) tcctggtcctccttcaacac; mouse *Cry2*, (forward) gcagagcctggttcaagc and (reverse) gccactggatagtgctctgg; mouse *Sec23b*, (forward) tgaccaaactggacttctgga and (reverse) aaagaatctcccatcaccatgt; mouse *Son*, (forward) ttccgggaaatacaacagga and (reverse) gggtggatttgtttcaccat; mouse *Manf*, (forward) gacagccagatctgtgaactaaaa and (reverse) tttcacccggagcttcttc; mouse *Manf* pre-mRNA, (forward) AGGGTATGCAGAGATGGTAGA and (reverse) GATCTGTGAGAAGCTGAAGAAGA; mouse *Hyou1*, (forward) GAGGCGAAACCCATTTTAGA and (reverse) GCTCTTCCTGTTCAGGTCCA; mouse *Hyou1* pre-mRNA, (forward) ACCGCTACAGCCATGATTT and (reverse) ATCATCTGGCAGGCACAC; mouse *Atf4*, (forward) CCACTCCAGAGCATTCCTTTAG and (reverse) CTCCTTTACACATGGAGGGATTAG; mouse *Atf6*, (forward) CATGAAGTGGAAAGGACCAAATC and (reverse) CAGCCATCAGCTGAGAATTAGA; mouse *Ire1*α, (forward) TCCTAACAACCTGCCCAAAC and (reverse) TCTCCTCCACATCCTGAGATAC; mouse 18*S* RNA, (forward) ctcaacacgggaaacctcac and (reverse) cgctccaccaactaagaacg; and mouse β*-actin*, (forward) aaggccaaccgtgaaaagat and (reverse) gtggtacgaccagaggcatac.

### ChIP and ChIP-seq

ChIP for SC35 was performed using anti-SC35 antibody (Abcam, ab11826) as previously described (*53*). Briefly, mouse liver samples were submerged in PBS + 1% formaldehyde, cut into small (~1 mm^3^) pieces with a razor blade, and incubated at room temperature for 15 min. Fixation was stopped by the addition of 0.125 M glycine (final concentration). The tissue pieces were then treated with a TissueTearer and, lastly, spun down and washed twice in PBS. Chromatin was isolated by the addition of lysis buffer, followed by disruption with a Dounce homogenizer. The chromatin was enzymatically digested with MNase. Genomic DNA (input) was prepared by treating aliquots of chromatin with ribonuclease (RNase) and proteinase K and heated for reverse cross-linking, followed by ethanol precipitation. Pellets were resuspended, and the resulting DNA was quantified on a NanoDrop spectrophotometer. An aliquot of chromatin (10 μg) was precleared with protein A agarose beads (Invitrogen). Genomic DNA regions of interest were isolated using 4 μg of antibody. Complexes were washed, eluted from the beads with SDS buffer, and subjected to RNase and proteinase K treatment. Cross-linking was reversed by incubation overnight at 65°C, and ChIP DNA was purified by phenol-chloroform extraction and ethanol precipitation. The DNA libraries were prepared at the University of Pittsburgh and sequenced at Gene by Gene Ltd. per the standard protocols. DNA libraries were prepared with an Ovation Ultralow V2 DNA-Seq library preparation kit (NuGen) using 1 ng of input DNA. The size selection for libraries was performed using SPRIselect beads (Beckman Coulter), and purity of the libraries was analyzed using the High Sensitivity DNA chip on Bioanalyzer 2100 (Agilent). The prepared libraries were pooled and sequenced using NovaSeq 6000 (Illumina), generating ~40 million 101–base pair single-end reads per samples. ChIP-qPCR for MEFs were essentially performed the same way as previously described with anti-SC35 (Abcam, ab11826) and anti-XBP1s antibody (BioLegend, 658802), except that the MEFs were directly fixed with 1% formaldehyde before subject to nuclei isolation and chromatin immunoprecipitation. The primers used for ChIP-qPCR are as follows: negative control region 1, (forward) GCAACAACAACAGCAACAATAAC and (reverse) CATGGCACCTAGAGTTGGATAA; negative control region 2, (forward) GCAGTATAACTTCTCACCCAAGT and (reverse) AACATGGTGTCTGTTTGCTTTC; *Xbp1* TSS region, (forward) GGCCACGACCCTAGAAAG and (reverse) GGCTGGCCAGATAAGAGTAG; *Xbp1* TTS region, (forward) CTTTCTCCACTCTCTGCTTCC and (reverse) ACACTAGCAAGAAGATCCATCAA; *Manf* TSS region, (forward) ACAGCAGCAGCCAATGA and(reverse) CAGAAACCTGAGCTTCCCAT; *Manf* TTS region, (forward) CAACCTGCCACTAGATTGAAGA and (reverse) AGGCATCCTTGTGTGTCTATTT; *Hyou1* TSS region, (forward) GACTTCGCAATCCACGAGAG and (reverse) GACTTCTGCCAGCATCGG; and *Hyou1* gene body region, (forward) TGGAAGAGAAAGGTGGCTAAAG and (reverse) TCCCAAGTGCTGGGATTAAAG.

### ChIP-seq analysis

Replicates were pooled at each time for subsequent ChIP-seq analysis. The sequences identified were mapped to the mouse genome (UCSC mm10) using BOWTIE function in Galaxy. Only the sequences uniquely mapped with no more than two mismatches were kept and used as valid reads. PCR duplicates were also removed. Peak calling was carried out by MACS2 (version 2.1.1.20160309) in Galaxy (options --mfold 5, 50 --pvalue 1e-4), on each ChIP-seq file against the input in XBP1*^Flox^* or XBP1*^LKO^* mice using the broad region function. To account for the different sequencing depths between samples, the signal files generated from MACS2 were reads per kilobase of transcript per million reads mapped normalized to sequencing depth. A total of 5365 genes with at least one peak identified in the gene body region (from TSS to TTS) in at least one CT in XBP1*^Flox^* mice were identified.

### RNA-seq quantification

RNA-seq data were as previously reported (*6*). Raw RNA-seq FASTQ files were analyzed by FastQC for quality control. Adaptors and low-quality reads were filtered by Trimmomatic (*54*). Then, the processed reads were aligned by HISAT2 (*55*) against mouse reference mm10. For gene-level intron/exon quantification, bedtools software (*56*) was used to collect and count reads that aligned to any intron/exon of the given gene. If one read spans across multiple exons of the same gene, then it will only be counted once. If one read spans intron/exon junction, then it will only be counted as intron. The intron/exon count was normalized by gene length and total reads for Fragments Per Kilobase of transcript per Million mapped reads normalization.

### Estimation of mRNA processing rate


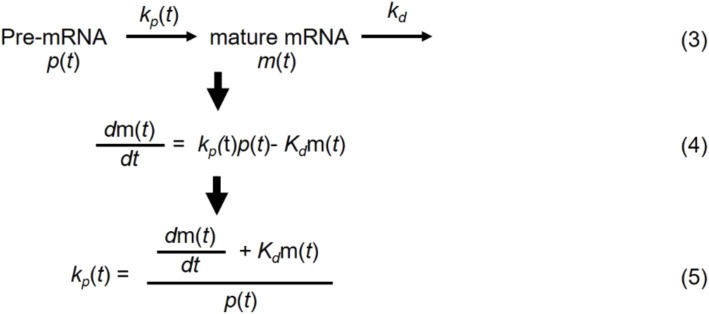


The mRNA processing rate was estimated by the simple kinetic model (Eq. 3), where pre-mRNA [*p*(*t*)] was converted to mature mRNA [*m*(*t*)] with the mRNA processing rate *k*_p_, which is also a function of time. We assume that the mature mRNA is subject to decay with a constant decay rate *k*_d_. Therefore, the mRNA processing rate *k_p_*(*t*) can be derived as Eq. 5. We used mRNA degradation rate for mouse genes reported in (*57*), and for gene without reported mRNA degradation rate, we used the mean value of 0.1 as a rough estimate. Because the original temporal gene expression data are fairly sparse (at 2-hour interval), to more accurately estimate the first derivative of mature mRNA [*m*(*t*)/*dt*], we performed a spline regression to obtain a more dense temporal dataset at 0.25-hour interval, and the first derivative at given time *t* is calculated as [*m*(*t + 0.25*) − *m*(*t*)]/0.25. Data analysis was performed in MATLAB and Excel.

### Identification of oscillations from temporal dataset

Three orthogonal methods were used to identify oscillations from temporal dataset. Periodogram power spectral density was generated using MATLAB with the pxx = periodogram (x) function. Eigenvalue/pencil analysis was performed in MATLAB with customarily written code as previously described (*4*, *28*). Criteria for circadian genes are period between 21 and 25 hours, and decay rate between 0.8 and 1.2; criteria for ~12-hour genes are period between 10.5 and 13.5 hours, and decay rate between 0.8 and 1.2. The FDR rate was calculated with a permutation-based method as previously described (*6*). RAIN analysis was performed as previously described in Bioconductor (3.4) (www.bioconductor.org/packages/release/bioc/html/rain.html) (*17*). For temporal SC35 ChIP-seq data in XBP1*^LKO^* mice, a polynomial detrend (*n* = 2) was first applied before being subjected to oscillation-identification algorithms. For all time-lapse microscopy data, the raw data, rather than the spline regression fit, were used to identify oscillations.

## Supplementary Materials

### This PDF file includes:

Figs. S1 to S17 Legends for tables S1 to S6 Legends for movies S1 to S3 References

## Notes

View/request a protocol for this paper from *Bio-protocol*.
