# Supplementary material for: Four-dimensional nuclear speckle phase separation dynamics regulate proteostasis
Source: Sci Adv. 2022 Jan 5;8(1):eabl4150. doi: 10.1126/sciadv.abl4150 (PMC8730402; doi:10.1126/sciadv.abl4150)
Supplement: Supplementary file 1 — Figs. S1 to S17 Legends for tables S1 to S6 Legends for movies S1 to S3 References [file sciadv.abl4150_sm.pdf]

Science Advances
AAAS

Supplementary Materials for

Four-dimensional nuclear speckle phase separation dynamics regulate proteostasis

William Dion, Heather Ballance, Jane Lee, Yinghong Pan, Saad Irfan, Casey Edwards, Michelle Sun, Jing Zhang, Xin Zhang, Silvia Liu, Bokai Zhu*

*Corresponding author. Email: bzhu@pitt.edu

Published 5 January 2022, *Sci. Adv.* **8**, eabl4150 (2022)
DOI: 10.1126/sciadv.abl4150

The PDF file includes:

Figs. S1 to S17
Legends for tables S1 to S6
Legends for movies S1 to S3
References

Other Supplementary Material for this manuscript includes the following:

Tables S1 to S6
Movies S1 to S3

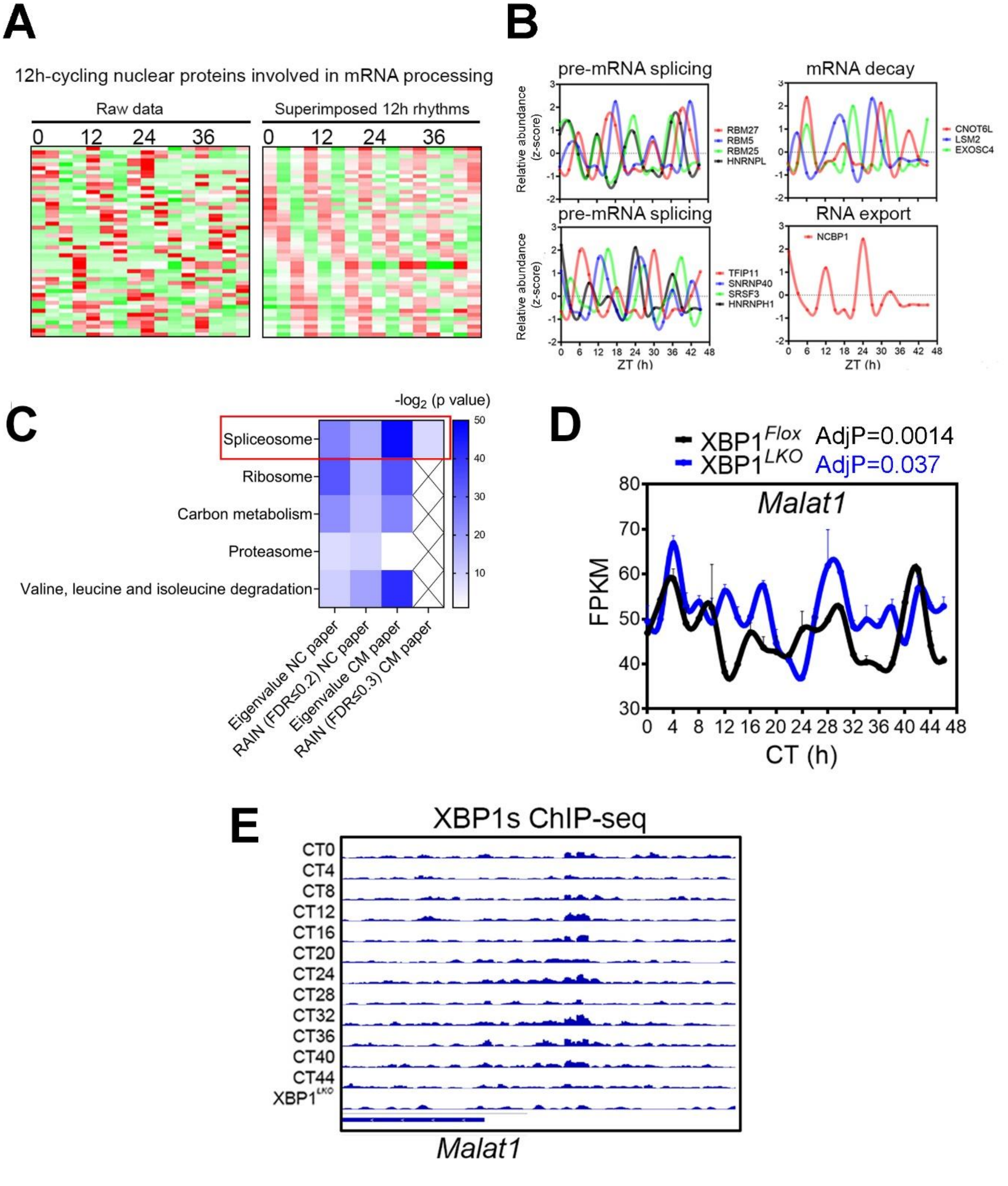

fig. S1. 12h rhythm of mRNA processing protein expression is prevalent in mouse liver. (**A**) Heat map of raw and superimposed 12h rhythms of nuclear proteins involved in mRNA metabolism as originally reported in (*9*). (**B**) Relative abundance of representative proteins at different zeitgeber time (ZT) as originally reported in (*9*). (**C**) GO analysis of 12h nuclear proteins reported in the *Nature Communications* (NC) (*9*) and *Cell Metabolism* (CM) (*10*) studies that are identified by either the eigenvalue/pencil or the RAIN method. (**D**) RNA-Seq data of *Malat1* expression in XBP1^{Flox} and XBP1^{LKO} mice (*Malat1* is intronless) with FDR adjusted p values by RAIN analysis shown. Data: Mean ± SEM. (**E**) Snapshot of XBP1s ChIP-Seq on *Malat1* promoter.

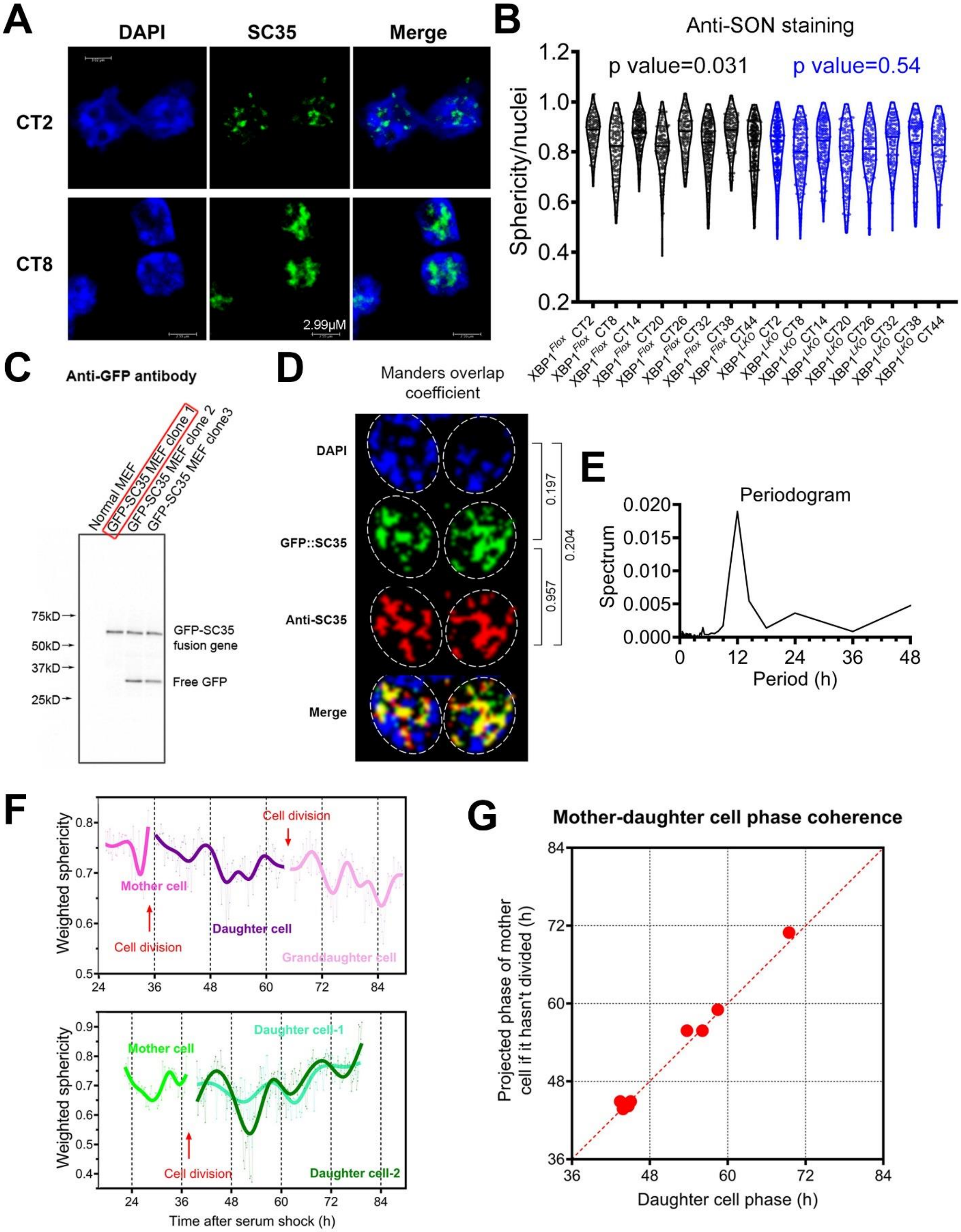

fig. S2. 12h rhythm of nuclear speckle morphology change is separate from the cell cycle. (**A**) Confocal immunofluorescence against SC35 co-stained with DAPI in the liver of XBP1^{Flox} at different CT. (**B**) Violin plot quantification of weighted sphericity of nuclear speckles in XBP1^{Flox}

and XBP1^{LKO} mice liver at different CT based upon anti-SON immunofluorescence signal. n=130~300 nuclei from 3 mice per CT. P values of exhibiting statistically significant 12h rhythms by RAIN analysis in XBP1^{Flox} and XBP1^{LKO} mice were also shown. (**C**) Anti-GFP western blot of normal as well three monoclonal lines of GFP::SC35 MEFs. Clone 1 is used in this study. (**D**) Confocal immunofluorescence against SC35 overlapped with GFP signal and co-stained with DAPI in GFP::SC35 MEFs. Manders coefficient between different signal are also shown. (**E**) Periodogram demonstrating cell-autonomous dominant 12h rhythmic nuclear speckle morphology change in MEFs, calculated from the raw average sphericity data in Fig. 1D. (**F**) Quantification of temporal sphericity from single GFP::SC35 MEFs at different times post serum synchronization. After each cell division, the temporal sphericity of daughter cells are continuously plotted after the mother cell. Narrow line: raw data, thick line: spline fit. (**G**) Scatter plot showing the phase relationship between the daughter cell and the mother cell if it hadn’t divided.

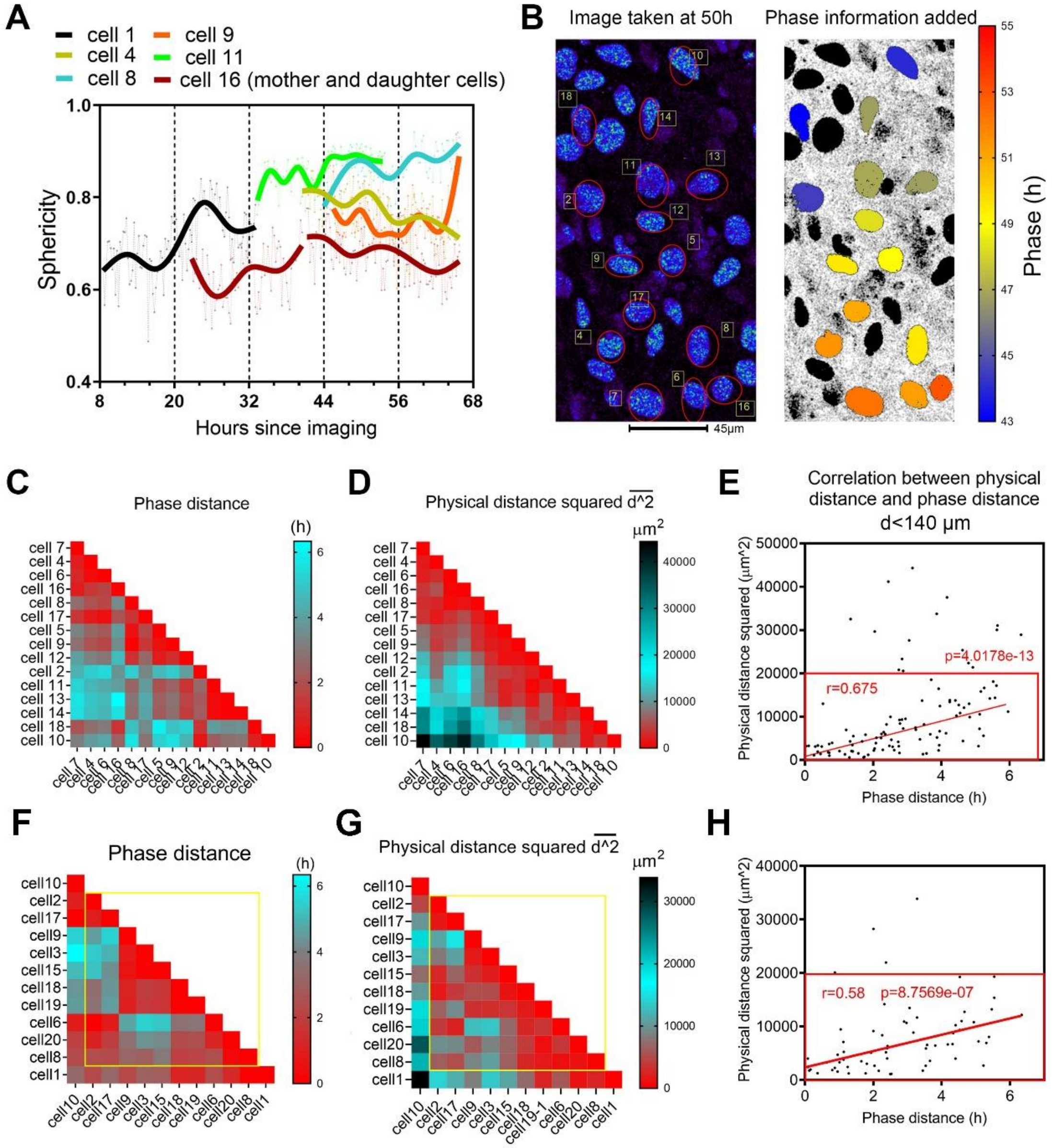

fig. S3. 12h rhythm of nuclear speckle morphology dynamics exhibits local coupling. (**A-E**) GFP::SC35 MEFs were subject to time lapse imaging without serum synchronization. Quantification of temporal sphericity from single MEFs (**A**). Representative image taken at hour 50 showing the physical location (left) and phase heat map (right) of different cells (**B**). Matrix showing the phase distance (**C**) and the square of physical distance (**D**) of different pairs of cells. Scatter plot of phase distance and the square of physical distance for different pairs of cells. Note that a positive correlation only exist for cells that are within 140 μm distance of each other. Pearson correlation coefficient *r* and p value that *r* is significantly larger than zero are shown for cells within 140 μm distance of each other (**E**). (**F-H**). An independent experiment showing similar data as C to E.

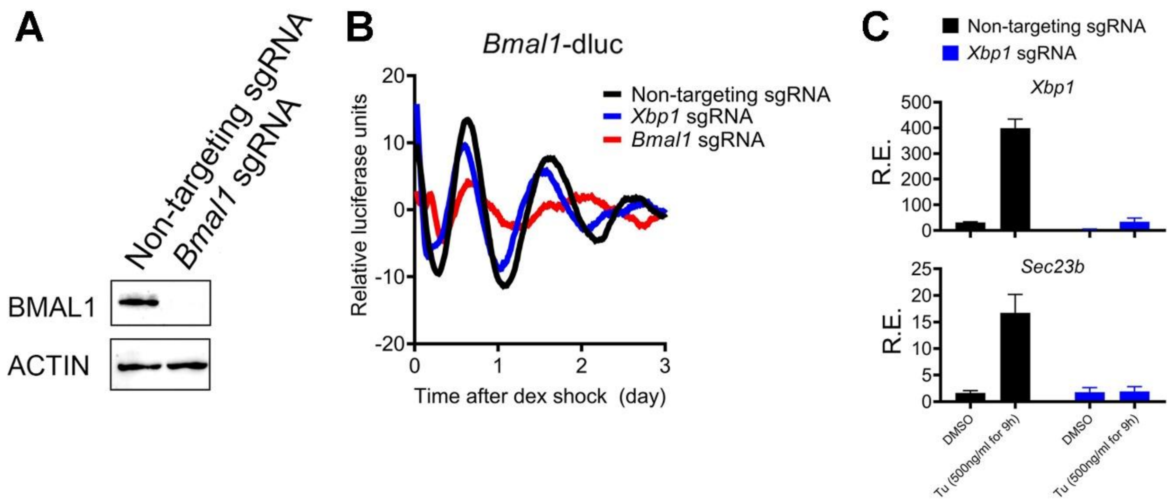

fig. S4. CRISPR/CAS9-mediated ablation of BMAL1 and XBP1 is successful in MEFs. (**A**) Western blot analysis of BMAL1 in GFP::SC35 MEFs expressing non-targeting or *Bmal1* sgRNA. (**B**) Real-time luminescence analysis of *Bmal1-dluc* MEFs post 100nM Dex treatment. Representative detrended traces of luminescence recordings from MEFs expressing non-targeting, *Bmal1* or *Xbp1* sgRNA. (**C**) qPCR analysis of *Xbp1* and UPR gene *Sec23b* in response to tunicamycin (Tu) treatment in GFP::SC35 MEFs expressing non-targeting or *Xbp1* sgRNA. The complete abolishment of *Sec23* induction by Tu indicates the functional ablation of XBP1 protein. Data: Mean ± SEM.

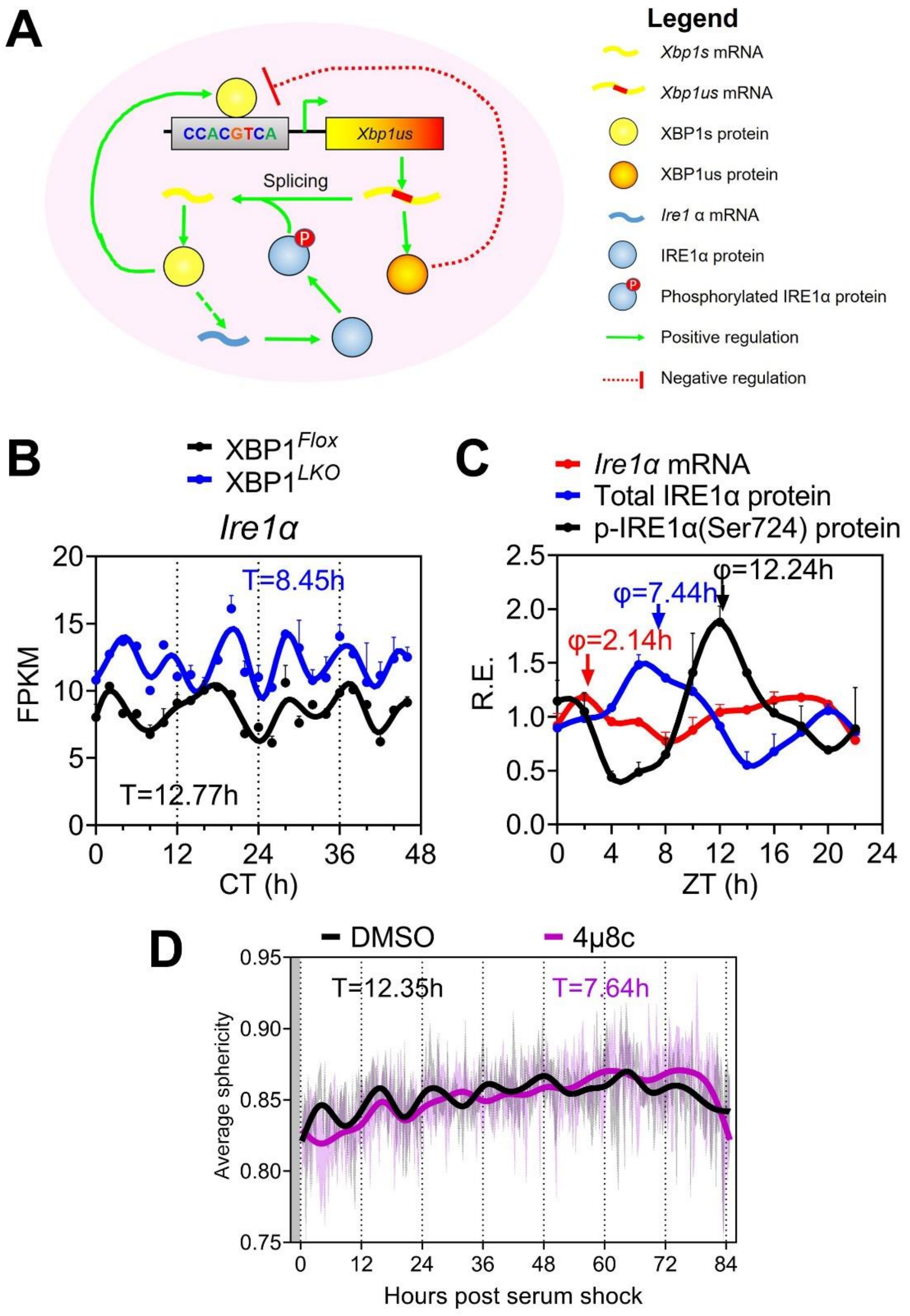

fig. S5. IRE1α inhibition impairs the 12h rhythm of nuclear speckle morphology change. (**A**) The regulatory network of 12h oscillator as previously proposed (*7*). XBP1s transcriptionally activates its own expression (*Xbp1us*) by binding to its own promoter (solid green arrow), whereas XBP1us has been previously shown to inhibit XBP1s transcriptional activity (*58*) (dashed red line). *Ire1α* mRNA exhibits an XBP1s-dependent 12h rhythm, thereby putting itself both downstream and upstream of XBP1s (solid green arrows). More importantly, since elevated IRE1α expression can increase *Xbp1* splicing in the absence of exogenous ER stress stimuli (*59*), mathematically,

it suggests that the 12h oscillator can, in theory, function without input on protein (mis)folding states from the ER. What remains unclear is whether XBP1s can directly transcriptionally regulate *Ire1α* mRNA via binding to its promoter or requires intermediate transcription factor(s) (dashed green arrow). (**B**) RNA-seq of hepatic *Ire1α* in XBP1^{FLOX} and XBP1^{LKO} mice, as reported in (*6*). (**C**) Quantification of hepatic total and phosphorylated IRE1α protein at different circadian time as reported in (*3*), overlaid with *Ire1α* RNA-seq data. Delayed phases from *Ire1α* mRNA to total IRE1α protein to phosphorylated IRE1α protein supports the model depicted in A. Data: Mean ± SEM. (**D**) Temporal sphericity of serum-synchronized GFP::SC35 MEFs treated with DMSO control or 10µM 4µ8c (average sphericity was calculated from each image that contains at least 15 cells, 3~4 images were taken per treatment; light area: mean ± SEM; solid line: spline fit), Gray areas indicates two hours of serum shock. Dominant periods from each group were calculated by the eigenvalue/pencil method.

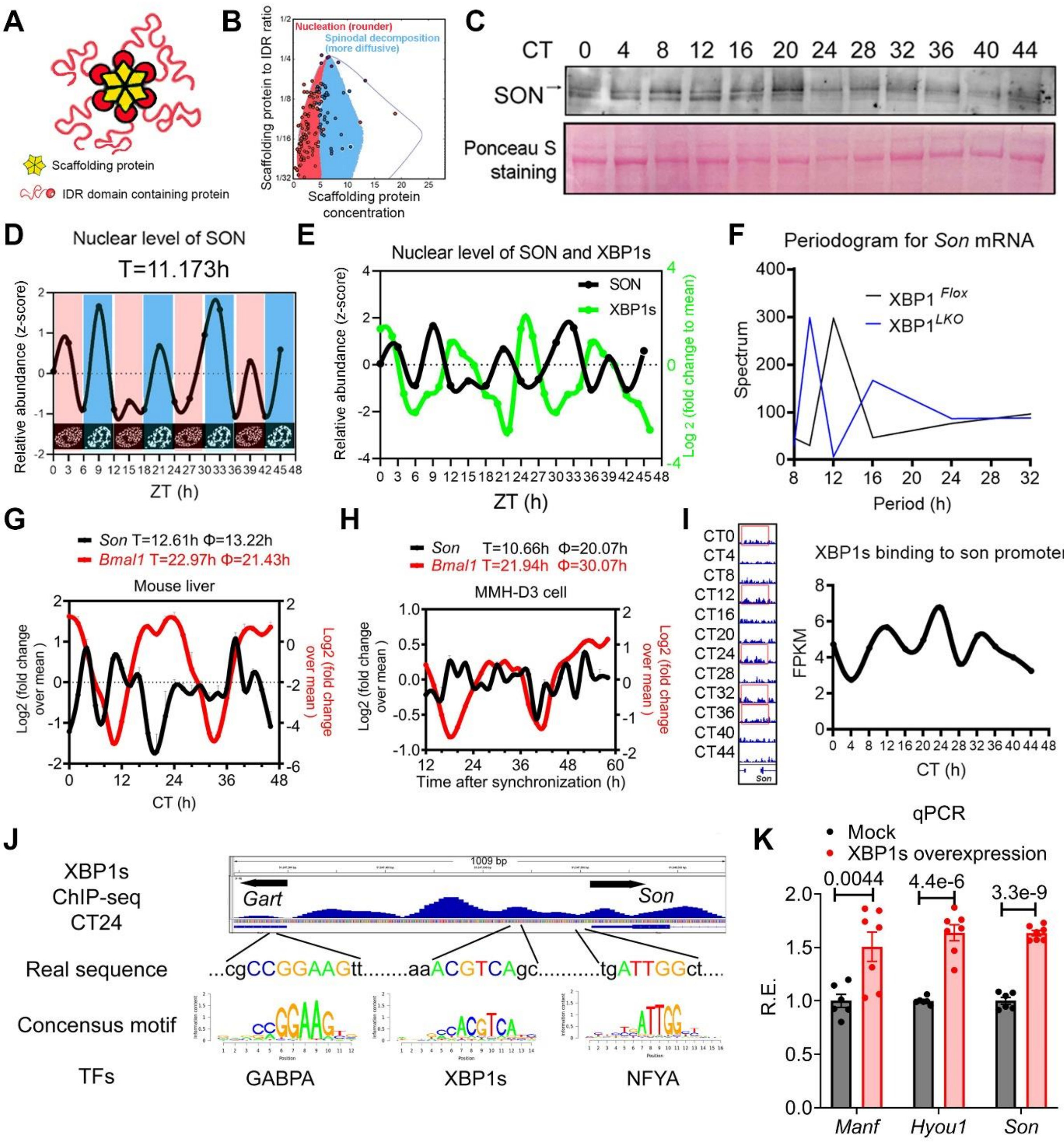

fig. S6. XBP1s regulates 12h rhythm of *Son* expression. (**A**) A cartoon showing the minimal components required for LLPS that include a multivalent 'scaffold' and multiple clients with IDR. (**B**) The phase diagram illustrating the conditions under which LLPS occurs via nucleation (red region) or spinodal decomposition (blue region). Under constant valency condition (the stoichiometry of clients to scaffold in the condensates as illustrated by the y-axis), increasing the concentration of scaffold protein will drive LLPS from nucleation to spinodal decomposition. (**C**) Additional western blot of hepatic SON protein at different CT with 4h resolution. (**D**) Relative abundance of hepatic SON at different zeitgeber time (ZT) superimposed with nuclear speckle morphology cartoons as reported in (*9*). (**E**) Relative abundance of hepatic SON protein superimposed with relative level of XBP1s protein quantified by western blot. (**F**) Periodogram of hepatic *Son* mRNA oscillation in XBP1^{Flox} and XBP1^{LKO} mice. (**G, H**) Log_2 transformed expression

of temporal *Son* and *Bmal1* in mouse liver (**G**) and MMH-D3 cells (**H**) with calculated period and phase by the eigenvalue/pencil method. (**I**) Snapshot (left) and quantification (right) of XBP1s ChIP-Seq signal at *Son* promoter at different CTs in XBP1^{Flox} mice as reported in (*6*). (**J**) Snapshot of XBP1s ChIP-Seq signal at CT24 at *Son* promoter as well as identified DNA binding motifs for GABPA, XBP1s and NFYA transcription factors. (**K**) qPCR analysis of *Son*, *Hyou1* and *Manf* expression in MEFs mock-transfected or transfected with Flag-XBP1s-PHAGE plasmid as previously described (*4*). Data: Mean ± SEM.

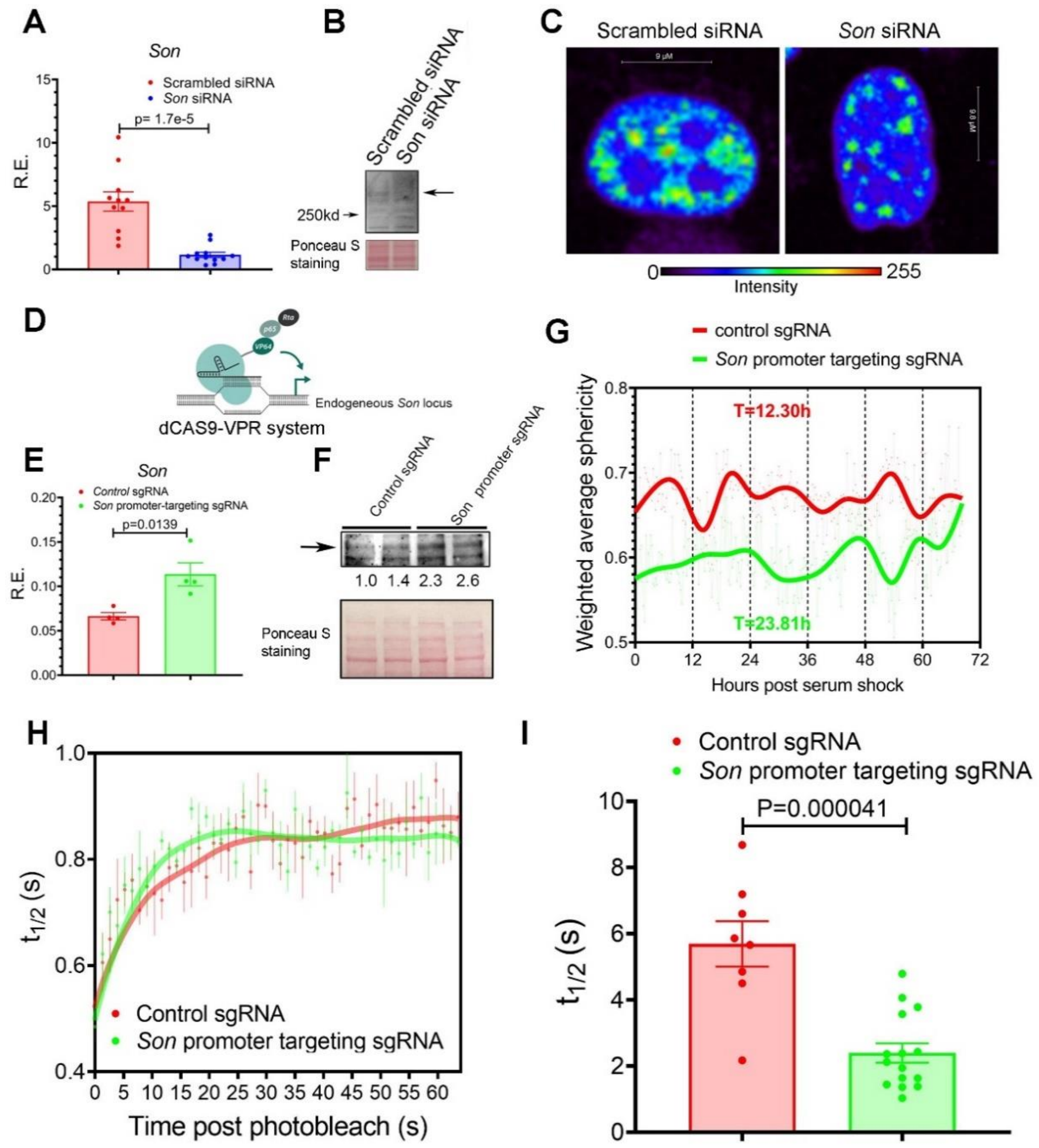

fig. S7. SON positively modulates nuclear speckle fluidity. (**A, B**) qPCR (**A**) and Western blot (**B**) of Son level in MEFs transfected with scrambled or *Son* siRNA. (**C**) Representative image of GFP::SC35 MEFs transfected with scrambled or *Son* siRNA. (**D-F**) Illustration of using the dCAS9-VPR system to transactivate endogenous *Son* gene expression (**D**). qPCR (**E**) and western blot (**F**) of Son level in MEFs stably expressing dCAS9-VPR and control or *Son* promoter-targeting sgRNA. (**G-I**) GFP::SC35 MEFs were stably expressing dCAS9-VPR and control or *Son* promoter-targeting sgRNA. After serum synchronization, weighted average sphericity were directly calculated from 10 to 30 cells at any given time with spline fit also shown, and periods were calculated by the eigenvalue/pencil method (**G**). FRAP analysis with representative recovery curve (**H**) (data showing quantification from 3 speckles per cell; mean ± SEM for each point; solid line: LOWESS fit) and quantified recovery half-life (**I**).

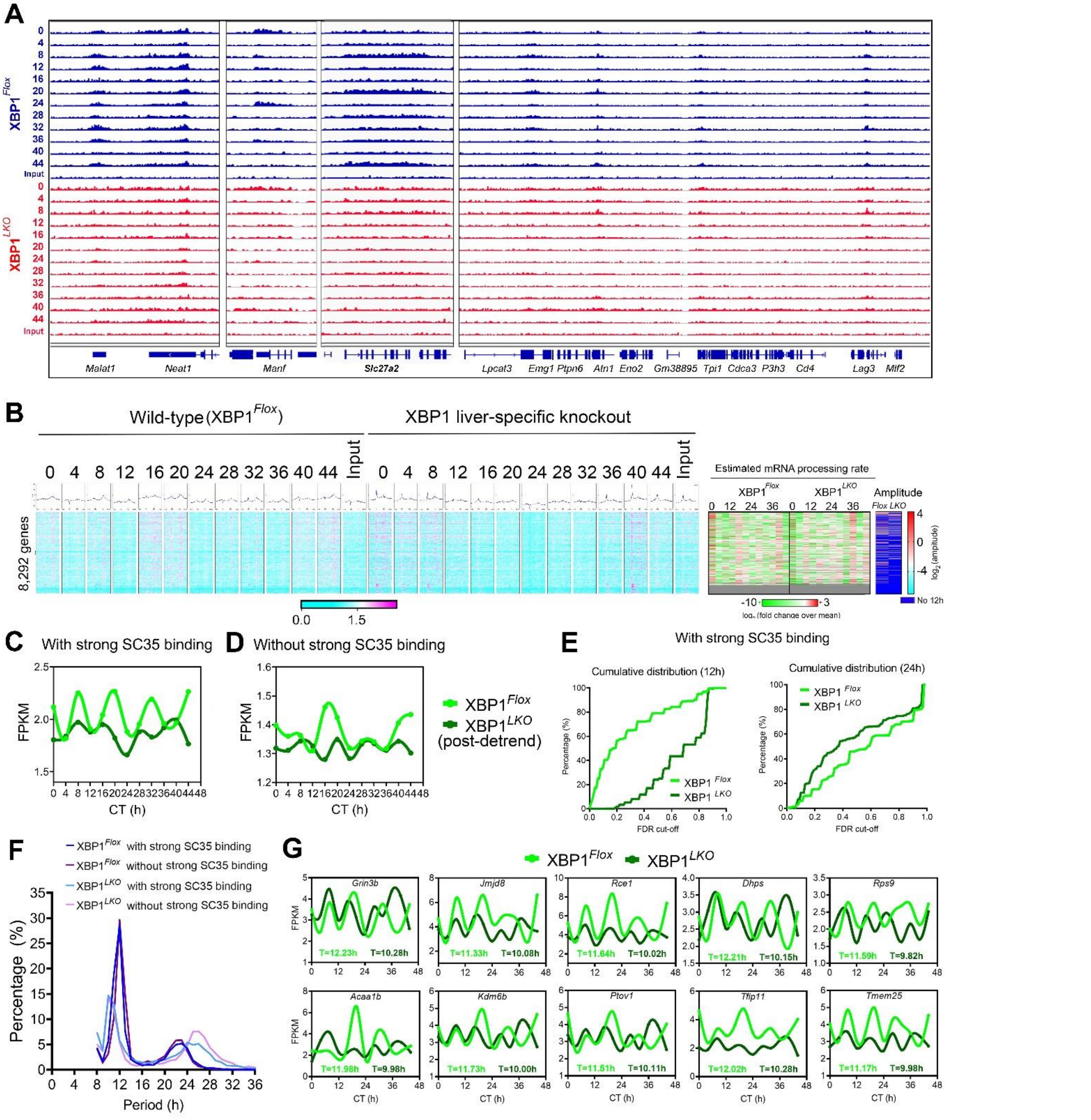

fig. S8. XBP1s regulates 12h nuclear speckle-chromatin interactions. (**A**) Snapshot of target genes selected for alignment of hepatic SC35 binding sites at different CTs in XBP1^{Flox} and XBP1^{LKO} mice. (**B**) Heat map of temporal SC35 as well as input signal for 8,292 genes that are below detection threshold for SC35 peak-calling algorithms in XBP1^{Flox} and XBP1^{LKO} mice from 10kb upstream of transcription start site (TSS) to 10kb downstream of transcription termination site (TTS) for each gene, aligned with the heat map of estimated mRNA processing rate (with amplitude for 12h rhythm) at different CT in XBP1^{Flox} and XBP1^{LKO} mice. (**C, D**) Quantification of

average integrated SC35 signal over gene bodies of 5,365 genes (**C**) and 8,292 genes (**D**) in XBP1^{Flox} and XBP1^{LKO} mice at different CTs. The quantification in XBP1^{LKO} mice is performed on data after polynomial detrend. (**E**) Cumulative distribution of the percentage of 12h (top) or 24h SC35 integrated signal (bottom) under different FDR cut-offs in both XBP1^{Flox} and XBP1^{LKO} mice from the RAIN analysis. Only those 5,365 genes with strong SC35 binding are included in the analysis. (**F**) Period distributions of all dominant oscillations (oscillation with the largest amplitude for each gene) of nuclear speckle-chromatin interactions uncovered by the eigenvalue/pencil method for 5,365 genes with strong SC35 binding and 8,292 genes without strong SC35 binding in both XBP1^{Flox} and XBP1^{LKO} mice. (**G**) Quantification of integrated SC35 signal over gene bodies for selected genes with ~12h nuclear speckle-chromatin interaction dynamics converted to ~10h ones with hepatic ablation of XBP1. The periods (calculated by the eigenvalue/pencil method) of nuclear speckle-chromatin interaction dynamics were shown for each gene in XBP1^{Flox} and XBP1^{LKO} mice.

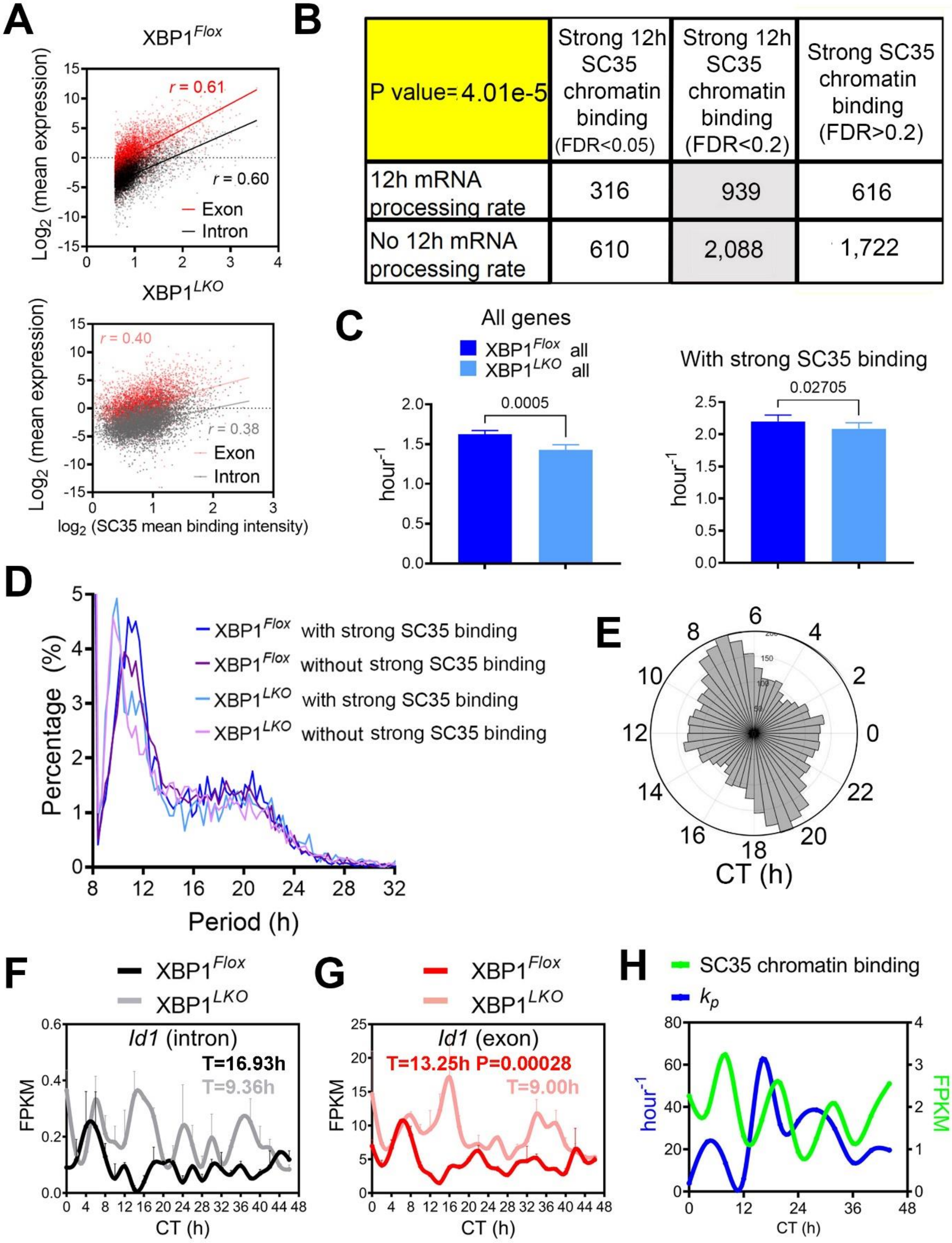

fig. S9. XBP1s regulates 12h mRNA processing rate. (**A**) Scatter plot showing the relationship between log_2 transformed values of pre (intron-mapping) and mature (exon-mapping) mRNA level and integrated SC35 ChIP-seq signal over gene bodies for 5,365 genes in XBP1^{Flox} (top) and

XBP1^{LKO} (bottom) mice. Pearson correlation coefficients *r* are further shown. (**B**) A table summarizing the number of genes with or without strong 12h SC35 signal and 12h mRNA processing rates under different FDR cut-off (from RAIN analysis) in XBP1^{Flox} mice. P value from chi-squared test indicating genes with 12h SC35 binding are also strongly associated with 12h mRNA processing rates. (**C**) Estimated mRNA processing rate for all (top) and 5,365 genes with strong SC35 signal (bottom) in both XBP1^{Flox} and XBP1^{LKO} mice. (**D**) Period distributions of all dominant oscillations (oscillation with the largest amplitude for each gene) of estimated mRNA processing rates uncovered by the eigenvalue/pencil method for 5,365 genes with strong SC35 binding and 8,292 genes without strong SC35 binding in both XBP1^{Flox} and XBP1^{LKO} mice. (**E**) Polar histogram demonstrating the phase distributions of 12h rhythmic mRNA processing rates for all genes in XBP1^{LKO} mice. (**F-H**) Temporal expression of *Id1* at the pre-mRNA, mature mRNA level in XBP1^{Flox} (**F**) and XBP1^{LKO} (**G**) mice, and integrated SC35 gene body signal and mRNA processing rate in XBP1^{Flox} mice (**H**). Data: Mean ± SEM.

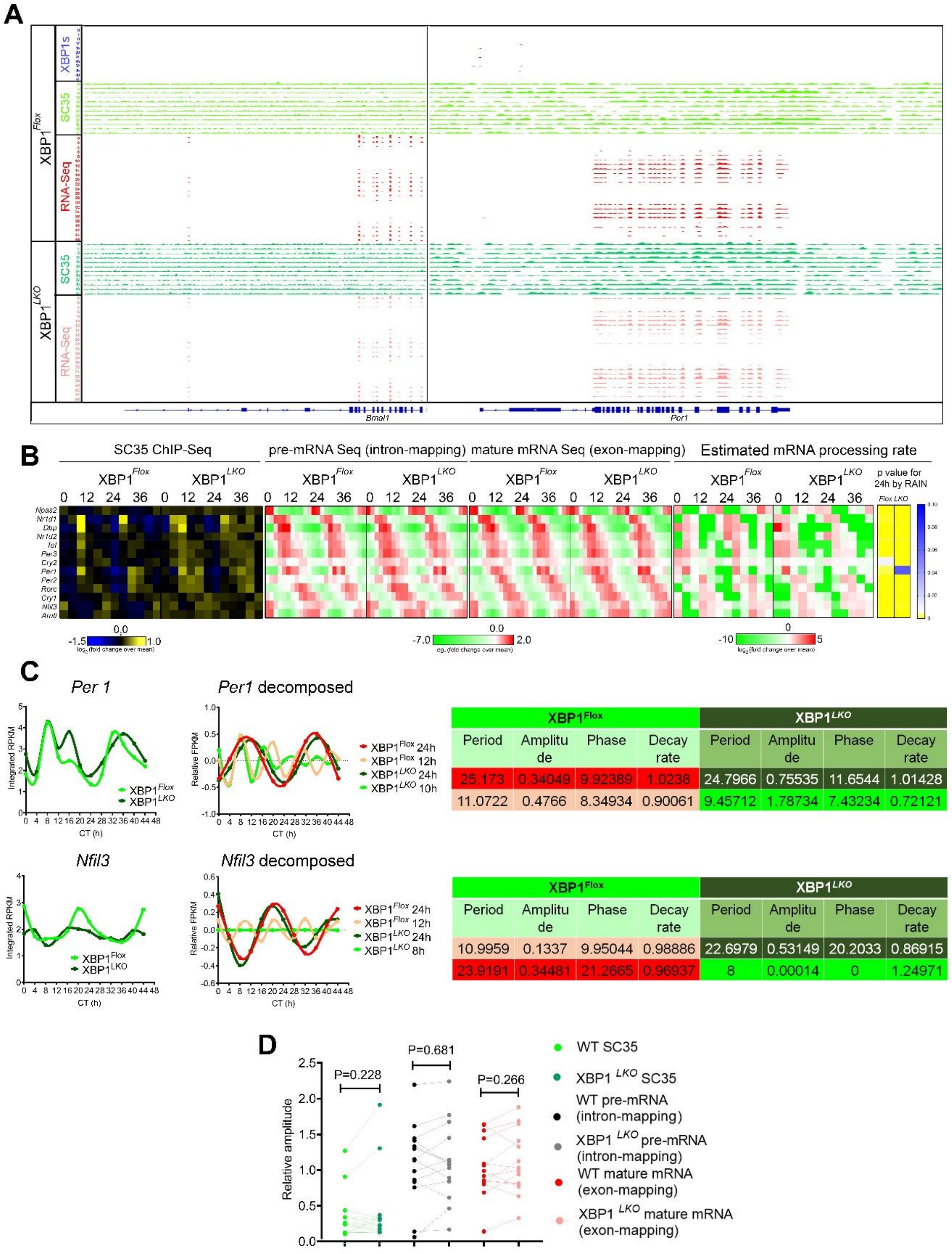

fig. S10. 12h nuclear speckle-chromatin interactions are dispensable for 24h core circadian clock gene expression. (**A**) Snapshot of core circadian clock genes selected for alignment of hepatic XBP1s, SC35 binding and RNA-Seq tracks at different CTs in XBP1^{Flox} and XBP1^{LKO} mice. (**B**) Heat maps of relative integrated SC35 binding signal over gene bodies, pre-mRNA and mature mRNA expression, and estimated mRNA processing rate (with p value for having a 24h

rhythm by RAIN) at different CT in XBP1^{Flox} and XBP1^{LKO} mice for core circadian clock genes. (**C**) Quantification of integrated SC35 signal over gene bodies for *Per1* and *Nfil3* (left), top two decomposed oscillations (middle) and the table showing the detailed parameters of different oscillations (right) by the eigenvalue analysis. Note the 24h components of rhythmic nuclear speckle-chromatin interaction are comparable between XBP1^{Flox} and XBP1^{LKO} mice, but the 12h component is abolished in XBP1^{LKO} mice. (**D**) Quantification of the relative amplitude of 24h SC35 ChIP-seq signal, and pre and mature mRNA oscillation for core circadian clock genes in XBP1^{Flox} and XBP1^{LKO} mice.

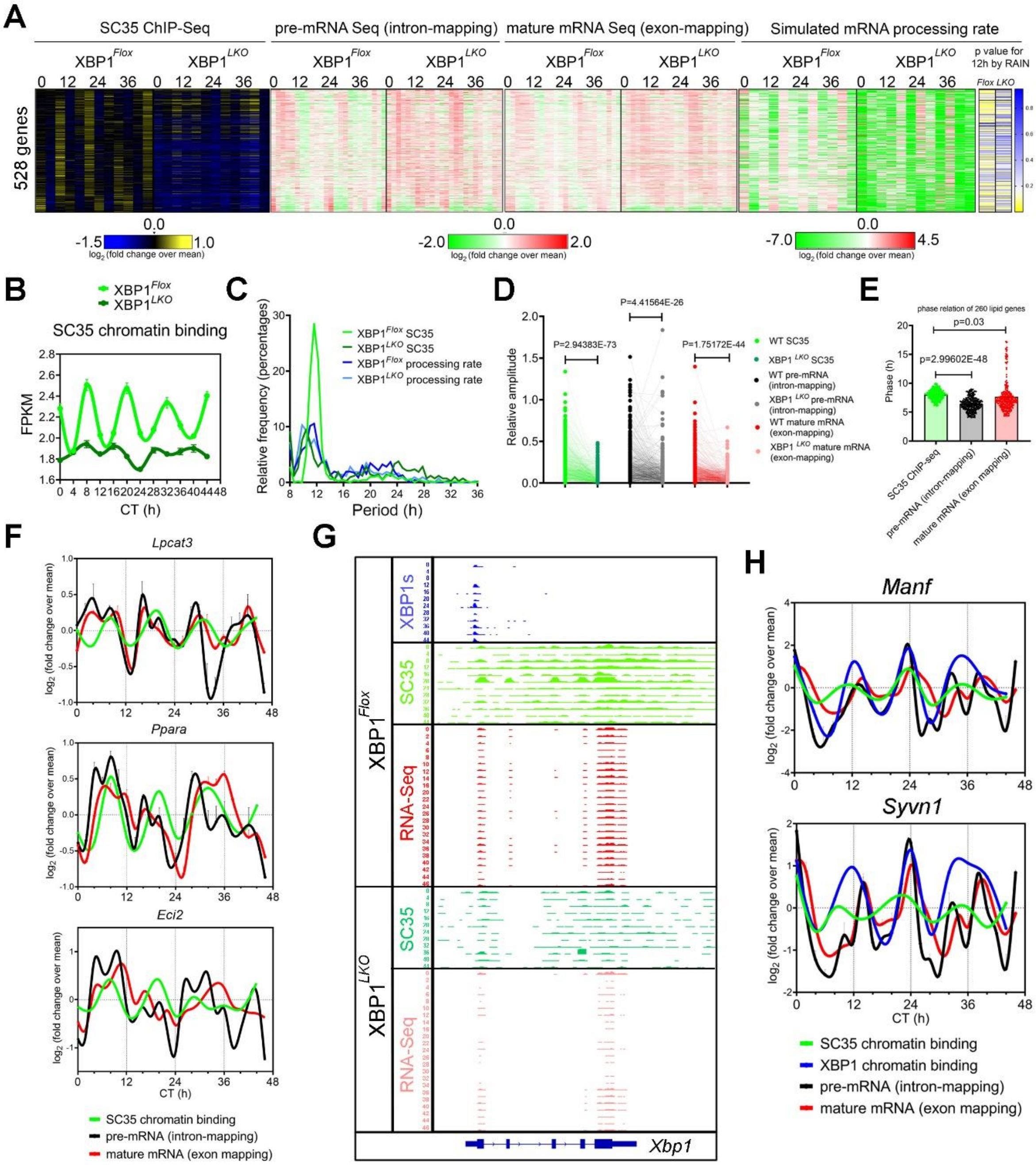

fig. S11. 12h nuclear speckle-chromatin interactions regulate UPR gene expression. (**A**) Heat maps of relative integrated SC35 binding signal over gene bodies, pre-mRNA and mature mRNA expression, and estimated mRNA processing rate (with p values for having a 12h rhythm by RAIN) at different CTs in XBP1^{Flox} and XBP1^{LKO} mice for 528 genes that exhibit very robust 12h rhythms of nuclear speckle-chromatin interaction as well as 12h rhythms of gene expression in XBP1^{Flox} mice. (**B**) Quantification of average integrated SC35 signal over gene bodies of 528 genes in XBP1^{Flox} and XBP1^{LKO} mice at different CTs. The quantification in XBP1^{LKO} mice is performed on data after polynomial detrend. (**C**) Period distributions of all dominant oscillations (oscillation with the largest amplitude for each gene) of estimated mRNA processing rates and

integrated SC35 signal over gene bodies uncovered by the eigenvalue/pencil method in XBP1^{Flox} and XBP1^{LKO} mice for 528 genes. (**D**) Quantification of relative amplitude of 12h SC35 ChIP-seq signal, and pre and mature mRNA oscillation for 528 genes in XBP1^{Flox} and XBP1^{LKO} mice. If no 12h rhythm was found, then the amplitude is deemed zero. (**E**) Quantification of the phases of 12h rhythmic SC35 signal, pre-mRNA and mature mRNA gene expression in 260 genes enriched in lipid metabolism and PPAR signaling in XBP1^{Flox} mice. Data: Mean ± SEM. (**F**) Relative temporal integrated SC35 binding signal over gene bodies, and pre and mature mRNA level for representative lipid metabolism genes in XBP1^{Flox} mice. (**G**) Snapshot of *Xbp1* locus for alignment of hepatic XBP1s, SC35 binding and RNA-Seq tracks at different CTs in XBP1^{Flox} and XBP1^{LKO} mice. (**H**) Relative temporal integrated SC35 binding signal over gene bodies, and pre and mature mRNA level for two UPR genes in XBP1^{Flox} mice.

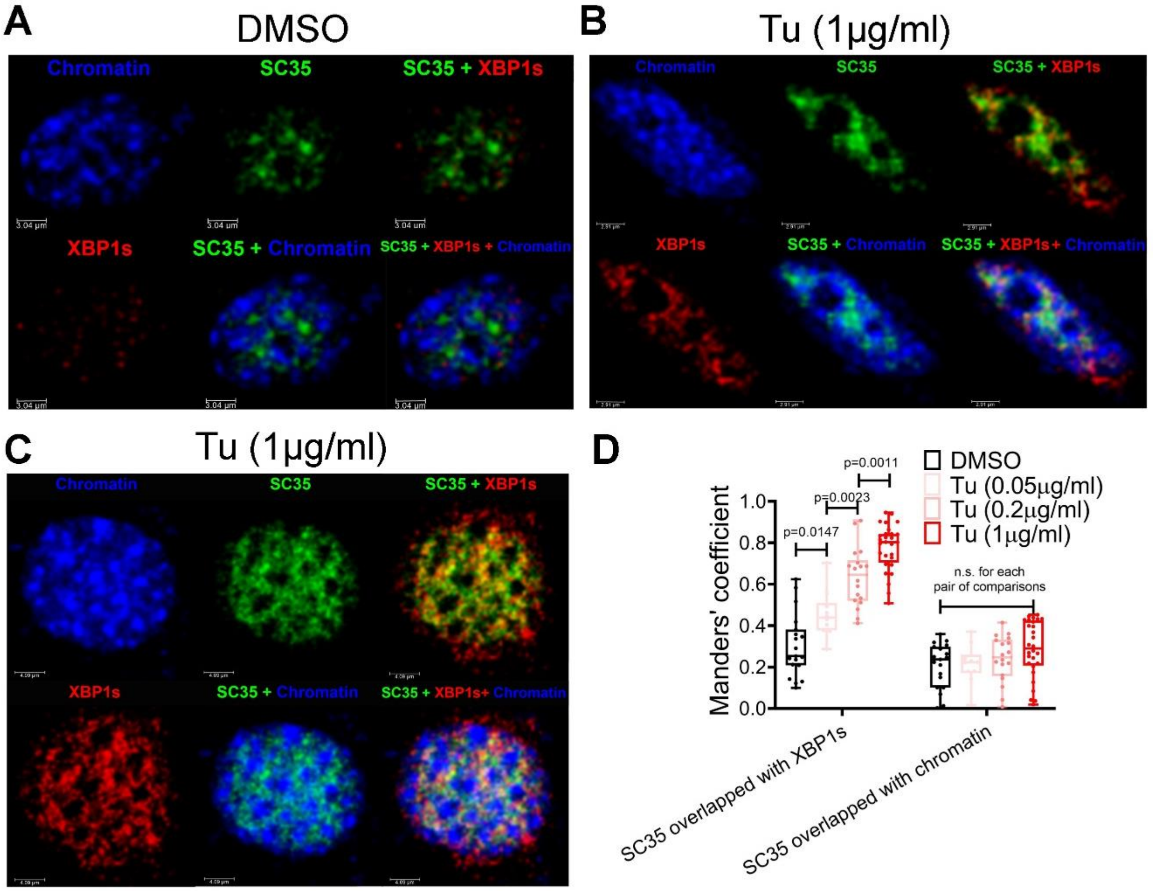

fig. S12. Co-localization of nuclear speckle and XBP1s is observed during UPR. Immunofluorescence of anti-XBP1s (red), GFP signal (green) from GFP::SC35 MEFs and DAPI nuclei staining (blue) as well as merged images of either two or all three channels in GFP::SC35 MEFs treated with increasing concentration of tunicamycin. Representative images from DMSO vehicle control group (**A**) and tunicamycin (1µg/ml) group (two representative images) (**B, C**), and Manders' coefficient quantification (**D**) of co-localization of SC35/XBP1s and SC35/DAPI signals. Box and whiskers plot showing minimum to maximum values.

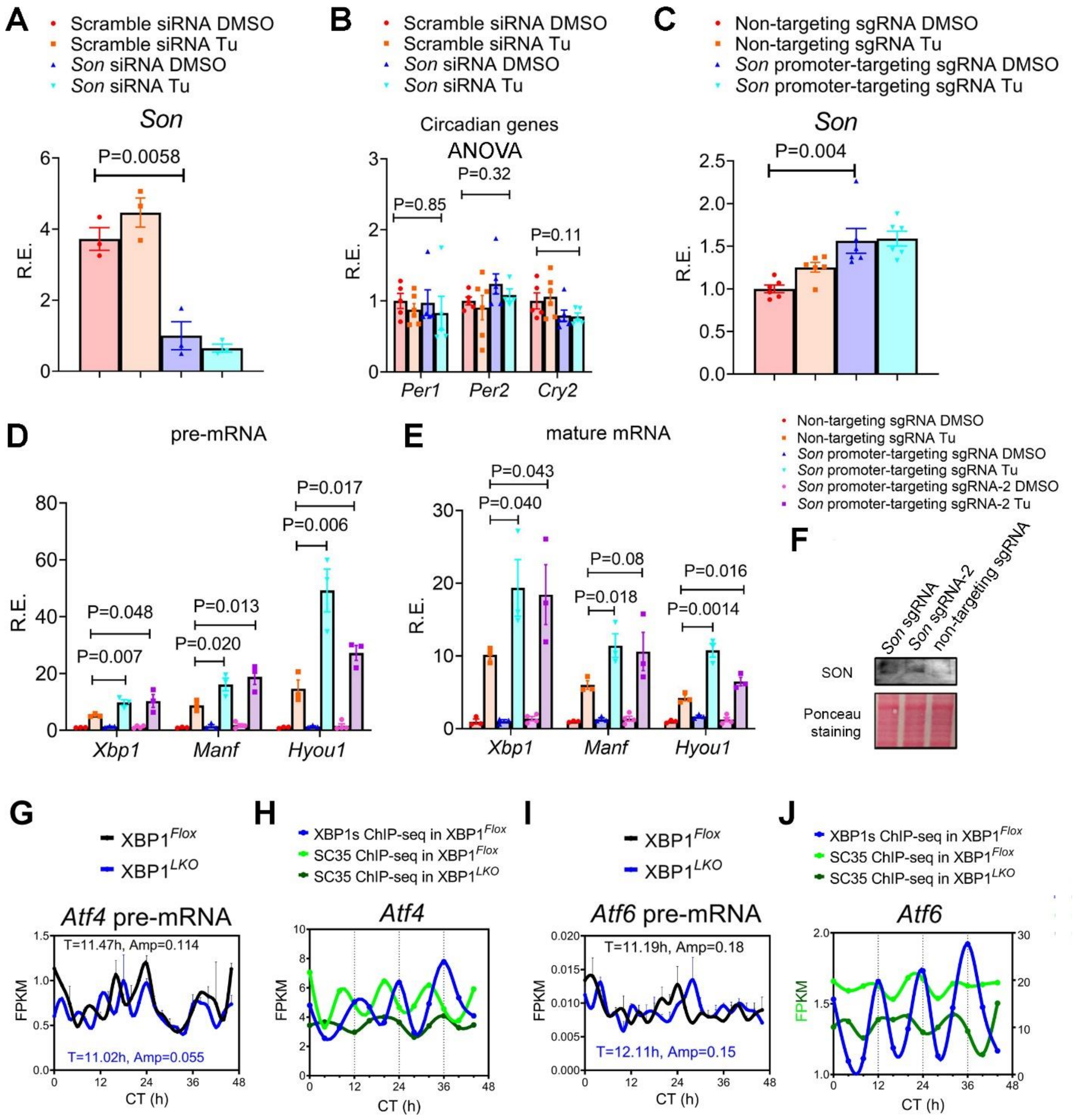

fig. S13. SON transcriptionally amplifies UPR. (**A-C**) MEFs with *Son* siRNA-mediated knocking down (**A, B**) or dCAS9-VPR-mediated stable overexpression of *Son* (**C**) were treated with 100ng/ml of tunicamcyin for 6 hours and qPCR analysis of different genes were performed. (**D-F**) dCAS9-VPR stably expressing MEFs were transiently transfected with non-targeting or two different *Son* promoter-targeting sgRNAs and treated with 100ng/ml of tunicamcyin for 6 hours. qPCR analysis of different genes was performed (**D, E**) and western blot of SON in DMSO group (**F**). (**G-J**) Temporal expression of *Atf4* (**G, H**) *and Atf6* (**I, J**) pre-mRNA level in XBP1^{Flox} and XBP1^{LKO} mice as reported in (*6*), XBP1s ChIP-seq signal in XBP1^{Flox} mice as reported in (*6*) and integrated SC35 gene body signal in XBP1^{Flox} and XBP1 ^{LKO} mice. Data: Mean ± SEM.

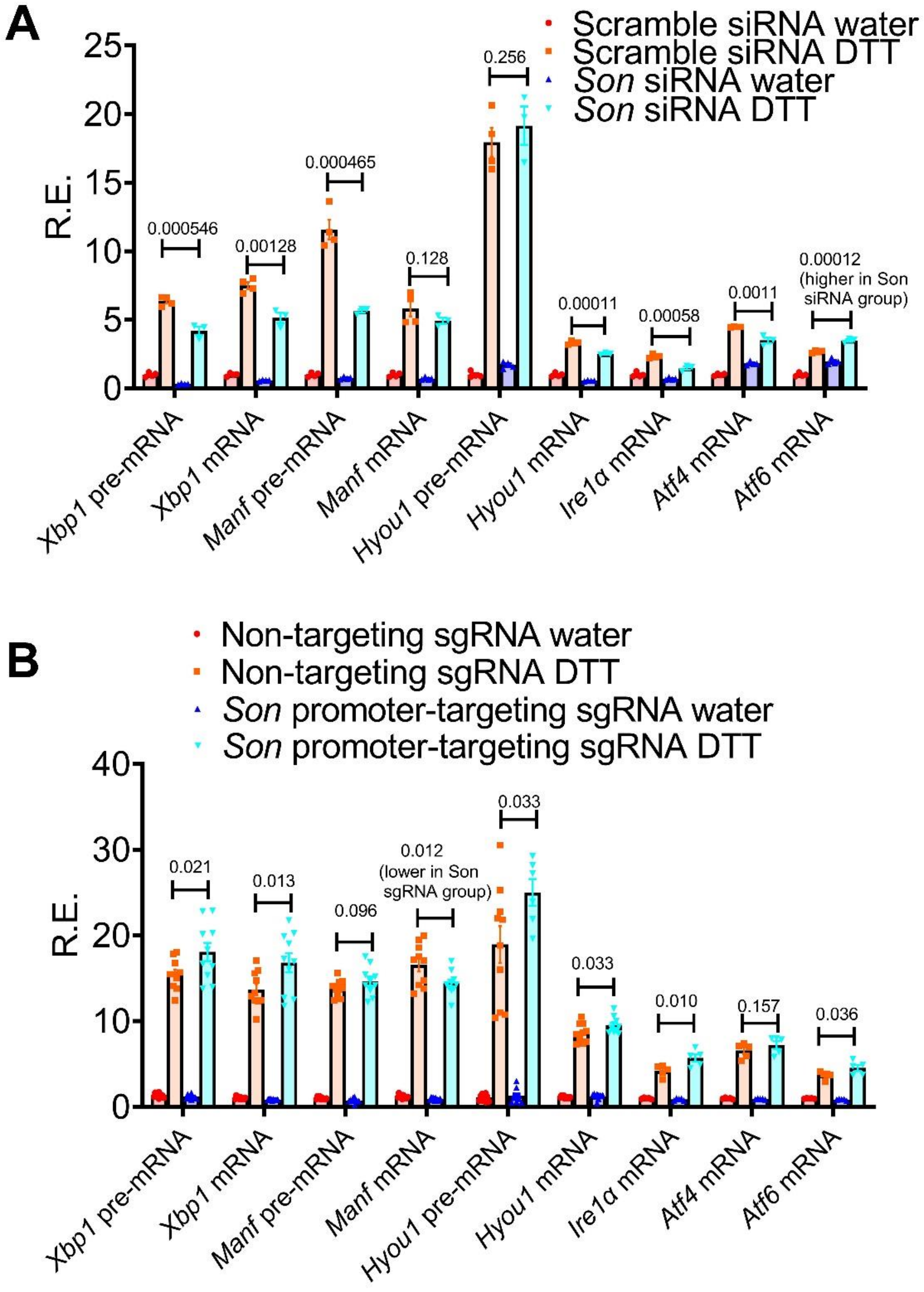

fig. S14. SON transcriptionally amplifies UPR in response to DTT. MEFs with *Son* siRNA-mediated knocking down (**A**) or dCAS9-VPR-mediated stable overexpression of *Son* (**B**) were treated with 1mM DTT for 4~5 hours and qPCR analysis of different genes were performed. Data: Mean ± SEM. Compared with tunicamycin-induced UPR, SON amplifies DTT-induced UPR with less potency, which is likely due to the fact that DTT can strongly activate the ATF6 branch of the UPR rapidly (*60*) and as previously demonstrated, SON has little effects on ATF6 expression.

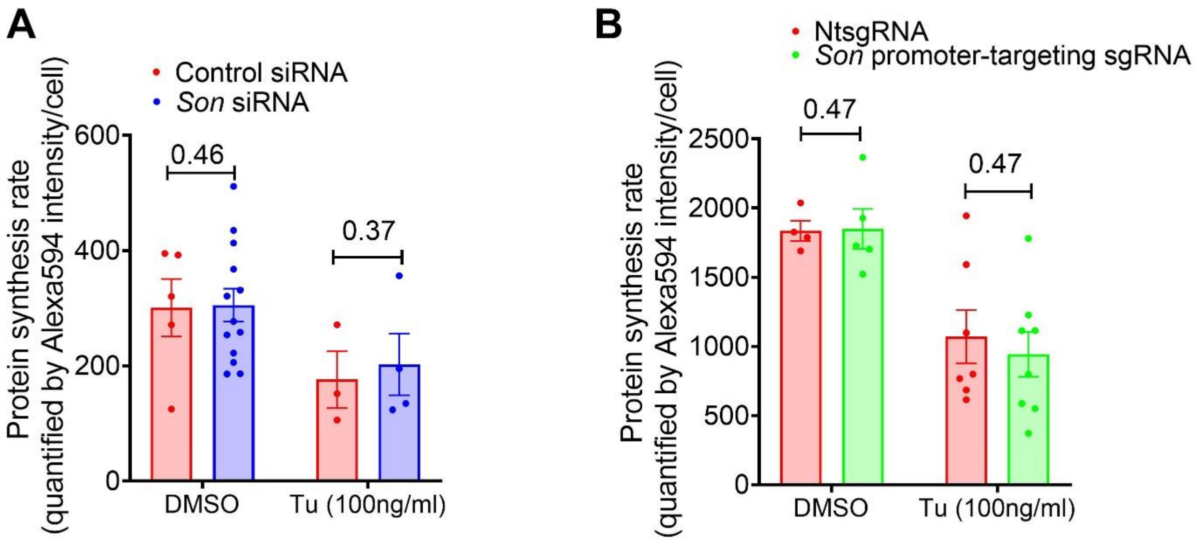

fig. S15. SON does not regulate global protein synthesis rate. Quantification of average protein synthesis rate per cell measured by the incorporation of Alexa594 labelled L-homopropargylglycine in MEFs that were transiently transfected with control or *Son* siRNAs (**A**) or stably expressing dCAS9-VPR and control or *Son* promoter-targeting sgRNAs (**B**) after 6h of DMSO or 100ng/ml Tu treatment. Data: Mean ± SEM.

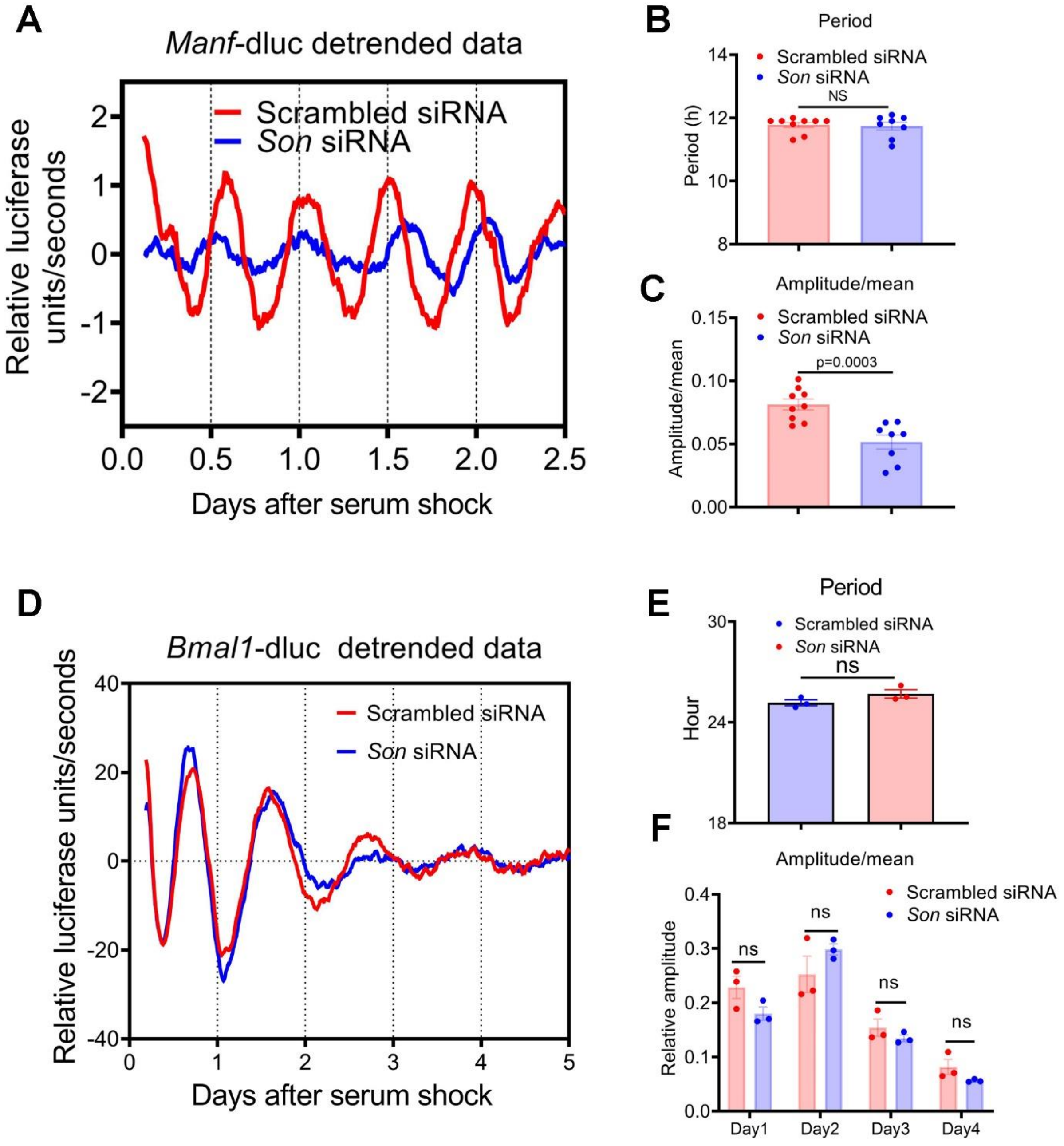

fig. S16. *Son* knocking-down impairs 12h ultradian, but not 24h circadian rhythm in MEFs. (**A-C**) *Manf*-dluc MEFs were transfected with control or *Son* siRNA. Detrend real-time luminescence (**A**), quantified period (**B**) and mean-normalized amplitude (**C**). (**D-F**) *Bmal1*-dluc MEFs were transfected with control or *Son* siRNA. Detrend real-time luminescence (**D**), quantified period (**E**) and mean-normalized amplitude (**F**). Data: Mean ± SEM.

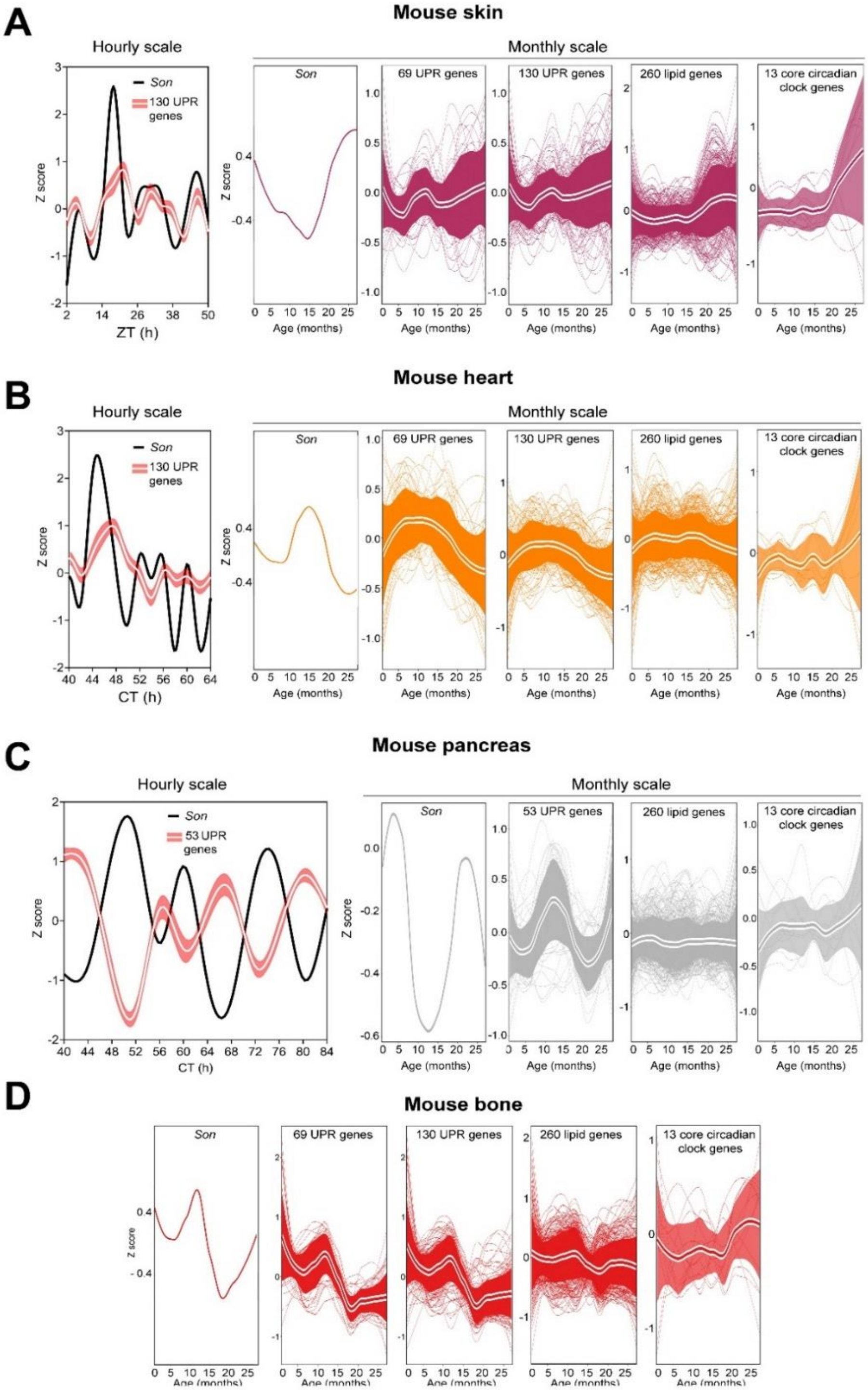

fig. S17. Correlative SON and UPR gene expression dynamics are observed across mouse life span. (**A-D**) Expression of *Son* and UPR genes in a 24~48h window (left) or across the entire mouse life span (right) in different tissues. For the mouse life span data, expression of 260 lipid genes and 13 core circadian clock genes are further shown. To the best of our knowledge, the *Son* or UPR diurnal gene expression status have not been reported in mouse bone. Solid line: mean; shaded area: 95% confidence interval.

table S1. 12h cycling nuclear proteins in mouse liver.

Tab1 All oscillating nuclear proteins idenitifed by the eigenvalue/pencil method (decay rate between 0.8 and 1.2) from Wang et al. (*9*)

Tab 2 All ~12h (period between 10.5h and 13.5h) oscillating nuclear proteins identified by the eigenvalue/pencil method from Wang et al. (*9*)

Tab 3 RAIN analysis on ~12h oscillating nuclear proteins from Wang et al. (*9*)

Tab 4 List and expression of 12h proteins involved in mRNA metabolism, as shown in Figure S1A. from Wang et al (*9*)

Tab 5 All oscillating nuclear proteins idenitifed by the eigenvalue/pencil method (decay rate between 0.8 and 1.2) from Wang et al. (*10*)

Tab 6 All ~12h (period between 10.5h and 13.5h) oscillating nuclear proteins identified by the eigenvalue/pencil method from Wang et al. (*10*)

Tab 7 RAIN analysis on ~12h oscillating nuclear proteins from Wang et al.(*10*)

Tab 8 List and expression of 12h proteins involved in mRNA metabolism, as shown in Figure S1A. from Wang et al. (*10*)

table S2. SC35 ChIP-Seq quantification for 5,365 genes.

Integrated SC35 ChIP-Seq signal over gene bodies (from TSS to TTS) were quantified at different CT for $XBP1^{Flox}$ and $XBP1^{LKO}$ mice for the 5,365 genes. For $XBP1^{LKO}$ mice, quantifications before and after polynomial detrend are shown.

table S3. RAIN analysis for 12h and 24h rhythms of SC35 ChIP-seq signal for 5,365 genes.

Tab 1 12h rhythm identified by RAIN in $XBP1^{Flox}$ mice

Tab 2 12h rhythm identified by RAIN in $XBP1^{LKO}$ mice

Tab 3 24h rhythm identified by RAIN in $XBP1^{Flox}$ mice

Tab 4 24h rhythm identified by RAIN in $XBP1^{LKO}$ mice

table S4. Eigenvalue/pencil analysis of SC35 ChIP-seq signal for all hepatically-expressed genes.

Tab 1 5,365 genes above peak-calling detection threshold in $XBP1^{Flox}$ mice

Tab 2 5,365 genes above peak-calling detection threshold in $XBP1^{LKO}$ mice

Tab 3 8,292 genes below peak-calling detection threshold in $XBP1^{Flox}$ mice

Tab 4 8,292 genes below peak-calling detection threshold in $XBP1^{LKO}$ mice

table S5. FPKM values of RNA-Seq data for exon-mapping reads in $XBP1^{Flox}$ and $XBP1^{LKO}$ mice.

table S6. FPKM values of RNA-Seq data for intron-mapping reads in $XBP1^{Flox}$ and $XBP1^{LKO}$ mice.

movie S1. Time lapse imaging of nuclear speckle morphology change in single GFP::SC35 MEF. The video is 5 frames per second.

movie S2. FRAP video of GFP::SC35 MEF transfected with scrambled siRNA. The video is 5 frames per second.

movie S3. FRAP video of GFP::SC35 MEF transfected with *Son* siRNA. The video is 5 frames per second.

REFERENCES AND NOTES

1. B. Zhu, C. C. Dacso, B. W. O'Malley, Unveiling "Musica Universalis" of the cell: A brief history of biological 12-hour rhythms. *J. Endocr. Soc.* **2**, 727–752 (2018).

2. M. E. Hughes, L. DiTacchio, K. R. Hayes, C. Vollmers, S. Pulivarthy, J. E. Baggs, S. Panda, J. B. Hogenesch, Harmonics of circadian gene transcription in mammals. *PLOS Genet.* **5**, e1000442 (2009).

3. G. Cretenet, M. Le Clech, F. Gachon, Circadian clock-coordinated 12 Hr period rhythmic activation of the IRE1alpha pathway controls lipid metabolism in mouse liver. *Cell Metab.* **11**, 47–57 (2010).

4. B. Zhu, Q. Zhang, Y. Pan, E. M. Mace, B. York, A. C. Antoulas, C. C. Dacso, B. W. O'Malley, A cell-autonomous mammalian 12 hr clock coordinates metabolic and stress rhythms. *Cell Metab.* **25**, 1305–1319.e9 (2017).

5. H. Meng, N. M. Gonzales, D. M. Lonard, N. Putluri, B. Zhu, C. C. Dacso, B. York, B. W. O’Malley, XBP1 links the 12-hour clock to NAFLD and regulation of membrane fluidity and lipid homeostasis. *Nat. Commun.* **11**, 6215 (2020).

6. Y. Pan, H. Ballance, H. Meng, N. Gonzalez, S.-M. Kim, L. Abdurehman, B. York, X. Chen, Y. Schnytzer, O. Levy, C. C. Dacso, C. A. McClung, B. W. O’Malley, S. Liu, B. Zhu, 12-h clock regulation of genetic information flow by XBP1s. *PLOS Biol.* **18**, e3000580 (2020).

7. B. Zhu, Decoding the function and regulation of the mammalian 12h-clock. *J. Mol. Cell Biol.* **12**, 752–758 (2020).

8. R. Sriburi, S. Jackowski, K. Mori, J. W. Brewer, XBP1: A link between the unfolded protein response, lipid biosynthesis, and biogenesis of the endoplasmic reticulum. *J. Cell Biol.* **167**, 35–41 (2004).

9. Y. Wang, L. Song, M. Liu, R. Ge, Q. Zhou, W. Liu, R. Li, J. Qie, B. Zhen, Y. Wang, F. He, J. Qin, C. Ding, A proteomics landscape of circadian clock in mouse liver. *Nat. Commun.* **9**, 1553 (2018).

10. J. Wang, D. Mauvoisin, E. Martin, F. Atger, A. N. Galindo, L. Dayon, F. Sizzano, A. Palini, M. Kussmann, P. Waridel, M. Quadroni, V. Dulic, F. Naef, F. Gachon, Nuclear proteomics uncovers diurnal regulatory landscapes in mouse liver. *Cell Metab.* **25**, 102–117 (2017).

11. N. Saitoh, C. S. Spahr, S. D. Patterson, P. Bubulya, A. F. Neuwald, D. L. Spector, Proteomic analysis of interchromatin granule clusters. *Mol. Biol. Cell* **15**, 3876–3890 (2004).

12. D. L. Spector, A. I. Lamond, Nuclear speckles. *Cold Spring Harb. Perspect. Biol.* **3**, a000646 (2011).

13. J. Fei, M. Jadaliha, T. S. Harmon, I. T. S. Li, B. Hua, Q. Hao, A. S. Holehouse, M. Reyer, Q. Sun, S. M. Freier, R. V. Pappu, K. V. Prasanth, T. Ha, Quantitative analysis of multilayer organization of proteins and RNA in nuclear speckles at super resolution. *J. Cell Sci.* **130**, 4180–4192 (2017).

14. Y. Shin, Y. C. Chang, D. S. W. Lee, J. Berry, D. W. Sanders, P. Ronceray, N. S. Wingreen, M. Haataja, C. P. Brangwynne, Liquid nuclear condensates mechanically sense and restructure the genome. *Cell* **176**, 1518 (2019).

15. D. Bracha, M. T. Walls, M. T. Wei, L. Zhu, M. Kurian, J. L. Avalos, J. E. Toettcher, C. P. Brangwynne, Mapping local and global liquid phase behavior in living cells using photo-oligomerizable seeds. *Cell* **175**, 1467–1480.e13 (2018).

16. J. Kim, K. Y. Han, N. Khanna, T. Ha, A. S. Belmont, Nuclear speckle fusion via long-range directional motion regulates speckle morphology after transcriptional inhibition. *J. Cell Sci.* **132**, jcs226563 (2019).

17. P. F. Thaben, P. O. Westermark, Detecting rhythms in time series with RAIN. *J. Biol. Rhythms* **29**, 391–400 (2014).

18. P. Bhat, D. Honson, M. Guttman, Nuclear compartmentalization as a mechanism of quantitative control of gene expression. *Nat. Rev. Mol. Cell Biol.* **22**, 653–670 (2021).

19. A. Sharma, H. Takata, K. Shibahara, A. Bubulya, P. A. Bubulya, Son is essential for nuclear speckle organization and cell cycle progression. *Mol. Biol. Cell* **21**, 650–663 (2010).

20. A. Atwood, R. DeConde, S. S. Wang, T. C. Mockler, J. S. Sabir, T. Ideker, S. A. Kay, Cell-autonomous circadian clock of hepatocytes drives rhythms in transcription and polyamine synthesis. *Proc. Natl. Acad. Sci. U.S.A.* **108**, 18560–18565 (2011).

21. F. Rijo-Ferreira, J. S. Takahashi, Genomics of circadian rhythms in health and disease. *Genome Med.* **11**, 82 (2019).

22. H. Ballance, B. Zhu, Revealing the hidden reality of the mammalian 12-h ultradian rhythms. *Cell. Mol. Life Sci.* **78**, 3127–3140 (2021).

23. M. Sorek, Y. Schnytzer, H. W. Ben-Asher, V. C. Caspi, C. S. Chen, D. J. Miller, O. Levy, Setting the pace: Host rhythmic behaviour and gene expression patterns in the facultatively symbiotic cnidarian Aiptasia are determined largely by *Symbiodinium*. *Microbiome* **6**, 83 (2018).

24. Y. Schnytzer, N. Simon-Blecher, J. Li, H. W. Ben-Asher, M. Salmon-Divon, Y. Achituv, M. E. Hughes, O. Levy, Tidal and diel orchestration of behaviour and gene expression in an intertidal mollusc. *Sci. Rep.* **8**, 4917 (2018).

25. A. Chavez, M. Tuttle, B. W. Pruitt, B. Ewen-Campen, R. Chari, D. Ter-Ovanesyan, S. J. Haque, R. J. Cecchi, E. J. K. Kowal, J. Buchthal, B. E. Housden, N. Perrimon, J. J. Collins, G. Church, Comparison of Cas9 activators in multiple species. *Nat. Methods* **13**, 563–567 (2016).

26. Y. E. Guo, J. C. Manteiga, J. E. Henninger, B. R. Sabari, A. Dall'Agnese, N. M. Hannett, J. H. Spille, L. K. Afeyan, A. V. Zamudio, K. Shrinivas, B. J. Abraham, A. Boija, T. M. Decker, J. K. Rimel, C. B. Fant, T. I. Lee, I. I. Cisse, P. A. Sharp, D. J. Taatjes, R. A. Young,

Pol II phosphorylation regulates a switch between transcriptional and splicing condensates. *Nature* **572**, 543–548 (2019).

27. J. A. West, C. P. Davis, H. Sunwoo, M. D. Simon, R. I. Sadreyev, P. I. Wang, M. Y. Tolstorukov, R. E. Kingston, The long noncoding RNAs NEAT1 and MALAT1 bind active chromatin sites. *Mol. Cell* **55**, 791–802 (2014).

28. A. C. Antoulas, B. Zhu, Q. Zhang, B. York, B. W. O'Malley, C. C. Dacso, A novel mathematical method for disclosing oscillations in gene transcription: A comparative study. *PLOS ONE* **13**, e0198503 (2018).

29. J. Kim, N. Khanna, A. S. Belmont, Transcription amplification by nuclear speckle association. *bioRxiv* **2019**, 604298 (2019).

30. J. Wang, L. Symul, J. Yeung, C. Gobet, J. Sobel, S. Luck, P. O. Westermark, N. Molina, F. Naef, Circadian clock-dependent and -independent posttranscriptional regulation underlies temporal mRNA accumulation in mouse liver. *Proc. Natl. Acad. Sci. U.S.A.* **115**, E1916–E1925 (2018).

31. P. Walter, D. Ron, The unfolded protein response: From stress pathway to homeostatic regulation. *Science* **334**, 1081–1086 (2011).

32. J. Yu, T. Li, Y. Liu, X. Wang, J. Zhang, X. Wang, G. Shi, J. Lou, L. Wang, C. C. Wang, L. Wang, Phosphorylation switches protein disulfide isomerase activity to maintain proteostasis and attenuate ER stress. *EMBO J.* **39**, e103841 (2020).

33. Y. Liu, M. Fares, N. P. Dunham, Z. Gao, K. Miao, X. Jiang, S. S. Bollinger, A. K. Boal, X. Zhang, AgHalo: A facile fluorogenic sensor to detect drug-induced proteome stress. *Angew. Chem. Int. Ed. Engl.* **56**, 8672–8676 (2017).

34. N. Schaum, B. Lehallier, O. Hahn, S. Hosseinzadeh, S. E. Lee, R. Sit, D. P. Lee, P. M. Losada, M. E. Zardeneta, R. Pálovics, T. Fehlmann, J. Webber, A. McGeever, H. Zhang, D. Berdnik, W. Tan, A. Zee, M. Tan, A. Pisco, J. Karkanias, N. F. Neff, A. Keller, S. Darmanis,

S. R. Quake, T. Wyss-Coray, The murine transcriptome reveals global aging nodes with organ-specific phase and amplitude. *bioRxiv* 662254 (2019).

35. M. Geyfman, V. Kumar, Q. Liu, R. Ruiz, W. Gordon, F. Espitia, E. Cam, S. E. Millar, P. Smyth, A. Ihler, J. S. Takahashi, B. Andersen, Brain and muscle Arnt-like protein-1 (BMAL1) controls circadian cell proliferation and susceptibility to UVB-induced DNA damage in the epidermis. *Proc. Natl. Acad. Sci. U.S.A.* **109**, 11758–11763 (2012).

36. R. Zhang, N. F. Lahens, H. I. Ballance, M. E. Hughes, J. B. Hogenesch, A circadian gene expression atlas in mammals: Implications for biology and medicine. *Proc. Natl. Acad. Sci. U.S.A.* **111**, 16219–16224 (2014).

37. M. Perelis, B. Marcheva, K. Moynihan Ramsey, M. J. Schipma, A. L. Hutchison, A. Taguchi, C. B. Peek, H. Hong, W. Huang, C. Omura, A. L. Allred, C. A. Bradfield, A. R. Dinner, G. D. Barish, J. Bass, Pancreatic β cell enhancers regulate rhythmic transcription of genes controlling insulin secretion. *Science* **350**, aac4250 (2015).

38. A. S. Lyon, W. B. Peeples, M. K. Rosen, A framework for understanding the functions of biomolecular condensates across scales. *Nat. Rev. Mol. Cell Biol.* **22**, 215–235 (2021).

39. M. Mofatteh, F. Echegaray-Iturra, A. Alamban, F. Dalla Ricca, A. Bakshi, M. G. Aydogan, Autonomous clocks that regulate organelle biogenesis, cytoskeletal organization, and intracellular dynamics. *eLife* **10**, e72104 (2021).

40. D. S. W. Lee, N. S. Wingreen, C. P. Brangwynne, Chromatin mechanics dictates subdiffusion and coarsening dynamics of embedded condensates. *Nat. Phys.* **17**, 531–538 (2021).

41. A. W. Folkmann, A. Putnam, C. F. Lee, G. Seydoux, Regulation of biomolecular condensates by interfacial protein clusters. *Science* **373**, 1218–1224 (2021).

42. S. E. Hasenson, Y. Shav-Tal, Speculating on the roles of nuclear speckles: How RNA-protein nuclear assemblies affect gene expression. *Bioessays* **42**, e2000104 (2020).

43. K. A. Alexander, A. Coté, S. C. Nguyen, L. Zhang, O. Gholamalamdari, P. Agudelo-Garcia, E. Lin-Shiao, K. M. A. Tanim, J. Lim, N. Biddle, M. C. Dunagin, C. R. Good, M. R. Mendoza, S. C. Little, A. Belmont, E. F. Joyce, A. Raj, S. L. Berger, p53 mediates target gene association with nuclear speckles for amplified RNA expression. *Mol. Cell* **81**, 1666–1681.e6 (2021).

44. D. Gems, L. Partridge, Stress-response hormesis and aging: "That which does not kill us makes us stronger". *Cell Metab.* **7**, 200–203 (2008).

45. A. H. Lee, E. F. Scapa, D. E. Cohen, L. H. Glimcher, Regulation of hepatic lipogenesis by the transcription factor XBP1. *Science* **320**, 1492–1496 (2008).

46. O. Shalem, N. E. Sanjana, E. Hartenian, X. Shi, D. A. Scott, T. Mikkelson, D. Heckl, B. L. Ebert, D. E. Root, J. G. Doench, F. Zhang, Genome-scale CRISPR-Cas9 knockout screening in human cells. *Science* **343**, 84–87 (2014).

47. A. Chavez, J. Scheiman, S. Vora, B. W. Pruitt, M. Tuttle, E. P. R. Iyer, S. Lin, S. Kiani, C. D. Guzman, D. J. Wiegand, D. Ter-Ovanesyan, J. L. Braff, N. Davidsohn, B. E. Housden, N. Perrimon, R. Weiss, J. Aach, J. J. Collins, G. M. Church, Highly efficient Cas9-mediated transcriptional programming. *Nat. Methods* **12**, 326–328 (2015).

48. E. Stashi, R. B. Lanz, J. Mao, G. Michailidis, B. Zhu, N. M. Kettner, N. Putluri, E. L. Reineke, L. C. Reineke, S. Dasgupta, A. Dean, C. R. Stevenson, N. Sivasubramanian, A. Sreekumar, F. Demayo, B. York, L. Fu, B. W. O'Malley, SRC-2 is an essential coactivator for orchestrating metabolism and circadian rhythm. *Cell Rep.* **6**, 633–645 (2014).

49. T. Sakuma, S. Nakade, Y. Sakane, K.-I. T. Suzuki, T. Yamamoto, MMEJ-assisted gene knock-in using TALENs and CRISPR-Cas9 with the PITCh systems. *Nat. Protoc.* **11**, 118–133 (2016).

50. B. Zhu, C. Khozoie, M. T. Bility, C. H. Ferry, N. Blazanin, A. B. Glick, F. J. Gonzalez, J. M. Peters, Peroxisome proliferator-activated receptor β/δ cross talks with E2F and attenuates mitosis in HRAS-expressing cells. *Mol. Cell. Biol.* **32**, 2065–2082 (2012).

51. G. Koulouras, A. Panagopoulos, M. A. Rapsomaniki, N. N. Giakoumakis, S. Taraviras, Z. Lygerou, EasyFRAP-web: A web-based tool for the analysis of fluorescence recovery after photobleaching data. *Nucleic Acids Res.* **46**, W467–W472 (2018).

52. A. Malovannaya, R. B. Lanz, S. Y. Jung, Y. Bulynko, N. T. Le, D. W. Chan, C. Ding, Y. Shi, N. Yucer, G. Krenciute, B. J. Kim, C. Li, R. Chen, W. Li, Y. Wang, B. W. O'Malley, J. Qin, Analysis of the human endogenous coregulator complexome. *Cell* **145**, 787–799 (2011).

53. B. Zhu, L. A. Gates, E. Stashi, S. Dasgupta, N. Gonzales, A. Dean, C. C. Dacso, B. York, B. W. O'Malley, Coactivator-dependent oscillation of chromatin accessibility dictates circadian gene amplitude via REV-ERB loading. *Mol. Cell* **60**, 769–783 (2015).

54. A. M. Bolger, M. Lohse, B. Usadel, Trimmomatic: A flexible trimmer for Illumina sequence data. *Bioinformatics* **30**, 2114–2120 (2014).

55. D. Kim, B. Langmead, S. L. Salzberg, HISAT: A fast spliced aligner with low memory requirements. *Nat. Methods* **12**, 357–360 (2015).

56. A. R. Quinlan, I. M. Hall, BEDTools: A flexible suite of utilities for comparing genomic features. *Bioinformatics* **26**, 841–842 (2010).

57. L. V. Sharova, A. A. Sharov, T. Nedorezov, Y. Piao, N. Shaik, M. S. Ko, Database for mRNA half-life of 19 977 genes obtained by DNA microarray analysis of pluripotent and differentiating mouse embryonic stem cells. *DNA Res.* **16**, 45–58 (2009).

58. H. Yoshida, M. Oku, M. Suzuki, K. Mori, pXBP1(U) encoded in XBP1 pre-mRNA negatively regulates unfolded protein response activator pXBP1(S) in mammalian ER stress response. *J. Cell Biol.* **172**, 565–575 (2006).

59. H. Li, A. V. Korennykh, S. L. Behrman, P. Walter, Mammalian endoplasmic reticulum stress sensor IRE1 signals by dynamic clustering. *Proc. Natl. Acad. Sci. U.S.A.* **107**, 16113–16118 (2010).

60. J. B. DuRose, A. B. Tam, M. Niwa, Intrinsic capacities of molecular sensors of the unfolded protein response to sense alternate forms of endoplasmic reticulum stress. *Mol. Biol. Cell* **17**, 3095–3107 (2006).
